# Differential Effects of Sodium Butyrate and Lithium Chloride on Rhesus Monkey Trophoblast Differentiation

**DOI:** 10.1371/journal.pone.0135089

**Published:** 2015-08-12

**Authors:** Priyadarsini Kumar, Twanda L. Thirkill, Jennifer Ji, Louise H. Monte, Gordon C. Douglas

**Affiliations:** Department of Cell Biology and Human Anatomy, School of Medicine, University of California Davis, Davis, California, United States of America; Sanford Burnham Medical Research Institute, UNITED STATES

## Abstract

Trophoblast differentiation during early placental development is critical for successful pregnancy and aberrant differentiation causes preeclampsia and early pregnancy loss. During the first trimester, cytotrophoblasts are exposed to low oxygen tension (equivalent to~2%-3% O_2_) and differentiation proceeds along an extravillous pathway (giving rise to invasive extravillous cytotrophoblasts) and a villous pathway (giving rise to multinucleated syncytiotrophoblast). Interstitial extravillous cytotrophoblasts invade the decidua, while endovascular extravillous cytotrophoblasts are involved in re-modelling uterine spiral arteries. We tested the idea that sodium butyrate (an epigenetic modulator) induces trophoblast differentiation in early gestation rhesus monkey trophoblasts through activation of the Wnt/β-catenin pathway. The results show that syncytiotrophoblast formation was increased by butyrate, accompanied by nuclear accumulation of β-catenin, and increased expression of EnvV2 and galectin-1 (two factors thought to be involved in trophoblast fusion). Surprisingly, the expression of GCM1 and syncytin-2 was not affected by sodium butyrate. When trophoblasts were incubated with lithium chloride, a GSK3 inhibitor that mimics Wnt activation, nuclear accumulation of β-catenin also occurred but differentiation into syncytiotrophoblast was not observed. Instead the cells differentiated to mononucleated spindle-shaped cells and showed molecular and behavioral characteristics of endovascular trophoblasts. Another highly specific inhibitor of GSK3, CHIR99021, failed to induce endovascular trophoblast characteristics. These observations suggest that activation of the Wnt/β-catenin pathway correlates with both trophoblast differentiation pathways, but that additional factors determine specific cell fate decisions. Other experiments suggested that the differential effects of sodium butyrate and lithium chloride might be explained by their effects on TNFα production. The results provide valuable tools to manipulate trophoblast differentiation *in vitro* and to better understand the differentiation pathways that occur during early gestation.

## Introduction

Trophoblast differentiation during early placental development proceeds along an extravillous pathway (giving rise to invasive extravillous cytotrophoblasts) and a villous pathway (giving rise to multinucleated syncytiotrophoblast). Interstitial extravillous cytotrophoblasts invade the decidua, interacting with decidual cells, while endovascular extravillous cytotrophoblasts enter the uterine spiral arteries and remodel vessel walls. The origin of these different trophoblast lineages remains uncertain and it is not clear whether a single progenitor gives rise to all phenotypes or whether more than one kind of progenitor cell exists. James et al provided evidence in favor of two populations of progenitor cells for villous and extravillous trophoblasts, respectively [1],while others suggest a single bi-potential progenitor [2, 3].

The Wnt/β-catenin pathway is known to play a role in trophoblast differentiation along both the villous and extravillous pathways [4, 5]. Activation of the Wnt/β-catenin pathway in human trophoblasts is thought to upregulate the transcription factor GCM1 [6] which in turn upregulates the expression of the fusogenic proteins syncytin-1 and syncytin-2 [7]. Activation of the Wnt/β-catenin pathway also induces trophoblast invasion [5].

Sodium butyrate has diverse effects on cells including induction of differentiation, inhibition of proliferation, modulation of immune response, and inhibition of inflammation [8–13]. Most of these effects are likely the result of sodium butyrate-mediated inhibition of histone deacetylase (HDAC) activity [13]although other non-HDAC pathways may also be involved [14]. Sodium butyrate is known to activate the Wnt/β-catenin pathway [15]. Previous studies have reported that sodium butyrate increases hCG production in trophoblast-derived choriocarcinoma cells [16] but little is otherwise known about the effects of butyrate on normal trophoblasts.

In the present paper we tested the idea that sodium butyrate induces trophoblast differentiation in early gestation rhesus monkey trophoblasts through activation of the Wnt/β-catenin pathway. Although sodium butyrate induced syncytiotrophoblast formation and caused nuclear accumulation of β-catenin, another Wnt activator, lithium chloride (LiCl), failed to induce syncytiotrophoblast formation and instead induced extravillous trophoblast differentiation. Thus, the Wnt pathway is involved in both trophoblast differentiation pathways but additional factors determine specific cell fate decisions. Other experiments suggest that the differential effects of sodium butyrate and lithium chloride might be explained by their differential effects on TNFα production.

## Materials and Methods

### Animals

All procedures involving Rhesus monkeys (*Macaca mulatta*) were performed in accordance with the NIH Guide for the Care and Use of Laboratory Animals and under the approval of the University of California Davis, Animal Care and Use Committee. This species was chosen because of certain known similarities in development to that of the human and our knowledge of the implantation process in these species. Studies of this kind are not possible with first trimester human placental tissue.

Adult rhesus macaques were housed at the California National Primate Research Center (CNPRC) on the University of California Davis campus. Animals were caged with a 0600–1800 hours light cycle and at a temperature maintained at 25–27°C. Animals were allowed to socialize by being housed in pairs during the day from approximately 0800–1400 hours. Animals were fed a diet of Purina Monkey Chow and water ad libitum. Seasonal produce, seeds and cereal were offered as supplements for environmental enrichment.

Timed pregnancies were obtained by mating the animals (4–8 years old) twice, 2 days apart, at the anticipated time of ovulation based on records of previous menstrual cycles, with the second mating designated as day of pregnancy. On day 16 of pregnancy, the presence of a conceptus was confirmed by ultrasound diagnosis. Placentas and endometrial tissue was collected at 52–55 days of pregnancy by surgical embryectomy which involved removal of the entire conceptus and surrounding endometrial tissue. Prior to surgery animals were fasted overnight. Pre-anesthetic analgesics such as oxymorphone or buprenorphine were administered as deemed necessary by veterinary staff. Other pre-anesthetic medications such as atropine, ketamine or glycopyrrolate were also used as needed. After endotracheal intubation anesthesia was induced using inhalant isoflurane or constant rate infusion of propofol, ketamine, fentanyl, lidocaine or dexmedetomidine. Heart rate, respiratory rate, oxygen saturation, blood pressure, C0_2_, and breathing pattern were continuously monitored. After uterotomy, the placenta, fetus and associated membranes were separated from the endometrial wall and removed intact. After closure of the uterotomy, oxytocin was administered. Isoflurane or constant infusion anesthetics were turned off and the endotracheal tube was removed. The animal was returned to the recovery cage and monitored every 15 minutes until able to sit up. Post-surgery analgesics were administered for at least 48 h following the procedure. Depending on the condition of the animal as assessed by the veterinary staff, either narcotic (oxymorphone, butorphanol, fentanyl CRI) or non-narcotic (flunixin meglumine, ketoprofen, aspirin, acetaminophen, ibuprofen) analgesics were used. Animals were then evaluated daily by veterinary staff to decide whether treatment should be continued or discontinued. Animals were monitored daily for food intake, fecal consistency and output, hydration, and activity. Discomfort was scored daily for 3 days and sutures are assessed daily for 7 days post-surgery. Experimental treatments were only performed in vitro on isolated cells and tissues obtained from this surgical procedure. Since no experimental treatments of animals were involved, only post-operative discomfort was expected. No animals were sacrificed and no complications resulting from surgery were reported.

### Trophoblast Isolation

Four rhesus placentas were obtained at 52–55 days of gestation (full gestation in this species is 170 days) and used for cell isolations. Trophoblasts were isolated from rhesus placental villous tissue using a modification of serial trypsin digestion methods originally described for human trophoblasts [17, 18]. Our procedure differs from that of Tarrade et al [17] by using a lower concentration of trypsin and increasing the number of sequential digestions. This increased yield without compromising purity. Villous tissue was cut into small pieces using scissors, washed using PBS, and subjected to sequential trypsin digestions at 37°C. First, the tissue was incubated without shaking with 0.1% trypsin for 10 min. The supernatant (digest 1) was collected. The remaining tissue was digested a second time using 0.2% trypsin for 10 min again without agitation. The supernatant was collected (digest 2). The tissue was digested a third time using 0.2% trypsin for 30 min but this time with agitation. Agitation was stopped and the tissue fragments were allowed to settle to the bottom of the flask. The supernatant (digest 3) was collected as above. The remaining tissue was digested sequentially five more times exactly as the third digest. The supernatants were collected and fetal bovine serum (FBS) was added to a final concentration of 10%. The additional five digestions vastly increased cell yield with no loss in trophoblast purity (based on CK7 and vimentin staining—see Results). The supernatants were centrifuged at 1000 rpm for 5 min at 4°C and the cell pellets were resuspended in DMEM/ F12 medium supplemented with fetal bovine serum (10%), 1X Minimal Essential Amino Acids, L-glutamine, and 0.1% gentamycin). This is referred to in the text as “trophoblast medium”. Cells from digests 1–3 were pooled and termed fraction A. Cells from digests 4–8 were pooled (Fraction B). The cells were filtered through 4 layers of sterile gauze and 2 layers of sterile nylon mesh and then centrifuged at 1000 rpm for 5 min at 4°C. The pooled fractions were layered on top of a 65%/30% Percoll step gradient and centrifuged at 1600 rpm for 20 min. The band of cells at the interface was collected, washed and resuspended in trophoblast medium. The cells were cryopreserved in 10% DMSO. As described in Results all experiments in this paper were carried out using cells from Fraction B.

Characterization of the isolated trophoblasts was carried out by immunocytochemical staining for the trophoblast marker cytokeratin 7 (CK7) and the mesenchyme marker vimentin (Vim). Nuclei were stained using DAPI. Freshly isolated cells were plated into LabTek culture chamber slides and double-stained with the antibodies 24 h after plating. Images of double-stained cells were captured and superimposed using Adobe Photoshop to allow quantitation of total number of nuclei, total number of CK7+ cells, and the total number of Vim+ cells. Using these three sets of values, the numbers of CK7+Vim- cells, CK7+Vim+ cells, CK7-Vim+ cells and CK7-Vim- cells were derived and expressed as a percentage of the total number of cells. Cells from four different placentas were analyzed. For Fraction B 100–200 cells were counted per field of view. For some Fraction A samples less than 100 cells were counted per field of view. This was due to the low cell recovery and the poor plating efficiency in this fraction.

### Trophoblast Culture

Cells were maintained in trophoblast medium in a hypoxic glove box (Coy Laboratories) at 2% oxygen and 5% CO2. The concentration of oxygen was selected to mimic the approximate oxygen tension encountered by trophoblasts during the early first trimester [19, 20]. For any manipulations, the culture medium was equilibrated for 5 hours in the hypoxic chamber prior to addition to the cells. Sodium butyrate was purchased from Stemgent (Cambridge MA) and was added at a final concentration of 0.5mM on alternate days in freshly equilibrated medium for 7 days.

### Endothelial Cell Culture

Human uterine microvascular endothelial cells (UtMVECs, passage 3) were purchased from Lonza (Walkersville, MD, USA) and maintained in endothelial basal medium-2 (EBM-2,) supplemented with EGM-2MV Single-Quots (human recombinant epidermal growth factor, human fibroblast growth factor, vascular endothelial growth factor, ascorbic acid, hydrocortisone, human recombinant insulin-like growth factor, gentamicin, 5% fetal calf serum [FCS]) exactly as described by the manufacturer. Cells between passages 5–7 were used for experiments.

### Antibodies

The antibodies used along with their dilutions and sources are shown in Table 1.

**Table 1:** Antibodies used in this study.

Name	Type	Dilution	Conc.	Source	Catalog #
		Western Blot	IFA		
**CK7**	M	n/a	2.5 μg/ml	Millipore	MAB3226
**Vimentin**	R	n/a	1: 2000	*	
**E-Cadherin**	M	1: 500	1 μg/ml	Abcam	ab1416
**Galectin-1**	R	1: 1000	2 μg/ml	Cell Signaling	12936
**β-Catenin**	R	n/a	1 μg/ml	Cell Signaling	9581
**α5-Integrin**	R	1: 2000	n/a	SantaCruz	Asc-10729
**α6-Integrin**	R	1: 500	n/a	Cell Signaling	3750
**αV-Integrin**	R	1: 1000	n/a	Cell Signaling	4711
**VE-Cadherin**	R (mAb)	n/a	0.21 μg/ml	Cell Signaling	2500
**PECAM1**	M	1: 250	n/a	DakoCytomation	M0823
**NCAM1**	M	1: 500	n/a	Millipore	MAB2120Z
**GAPDH**	M	1: 2000	n/a	SantaCruz	sc-32233

*Provided by Dr Paul Fitzgerald, Dept. of Cell Biology and Human Anatomy, University of California Davis. M, mouse monoclonal; R, rabbit polyclonal. n/a, not applicable since antibody was not used for that application.

### Immunocytochemistry

For immunofluorescence staining, adherent cells on 2-chamber glass LabTek culture slides were fixed with ice-cold 3.7% paraformaldehyde for 5 min and permeabilized using 0.2% Triton X-100. The slides were then blocked in PBS containing 0.2% gelatin and incubated overnight at 4°C with primary antibodies. The slides were then washed three times using PBS/gelatin and incubated with the appropriate AlexaFluor-conjugated secondary antibodies for 30 min. Nuclei were stained using 4',6-diamidino-2-phenylindole (DAPI). The slides were coverslipped using glycerol-based medium containing anti-fade reagent and viewed using a widefield microscope. Controls consisted of using matched mouse or rabbit immunoglobulin in place of the primary antibodies. Identical image capture settings were used for test and control samples. Captured images were pseudocolorized using Adobe Photoshop.

### Assessment of Syncytiotrophoblast Formation

The formation of multinucleated syncytiotrophoblast was assessed as we have described previously [21–24]. Trophoblasts were cultured in 2-chamber glass LabTek slides (Thermo-Scientific, USA). Specific treatments and incubation times are given in Results. The cells were then fixed and permeabilized using 3.7% formaldehyde/0.2% triton X-100 after which they were incubated in PBS/gelatin. Cell-cell junctions and nuclei were revealed by staining cultures simultaneously with a mouse monoclonal anti-E-Cadherin antibody (Abcam, Inc., USA) and 4',6-diamidino-2-phenylindole (DAPI) [25], respectively. The primary anti-E-cadherin antibody was detected using AlexaFluor-488-labeled goat anti-mouse secondary antibody (Invitrogen, USA). The slides were mounted using glycerol vinyl alcohol (GVA) aqueous mounting medium (Zymed, USA) and examined using an epifluorescence microscope. Images were captured using an Optronics DEI750 CCD camera and Q imaging software. Identical exposure settings were used for all captured images. Total numbers of nuclei (T) from three random fields per well were counted (200–400 nuclei per field of view). The total number (S) of syncytia (cells containing three or more nuclei) and the total number of nuclei in syncytia (N) were also counted. A Fusion Index (FI) was calculated using the formula (N-S/T)x100.

### Real time PCR

Total RNA was extracted from trophoblast cells using RNeasyplus kit (Qiagen) and cDNAs were obtained by reverse transcription (RT) using SuperscriptII reverse transcriptase (Life technologies, Grand Island NY). Real-time RT-PCR experiments were performed in triplicate using SYBR Green chemistry using StepOneplus Real-time PCR system (Life Technologies, Grand Island NY). StepOneplus software was used to calculate ΔCt values normalized against a GAPDH control. Fold change was calculated using the Livak ΔΔCt method [26]. The primers used are shown in Table 2.

**Table 2:** Primers used for qPCR.

Gene	forward primer	reverse primer
**GCM1**	purchased from Qiagen	
**Syncytin 2**	purchased from Qiagen	
**EnvV2** *	CATGACTTTGGAAAAGGAGG	ACCAAAGAGGAAAAGTAAGAGT
**Galectin-1**	AACAACCTGTGCCTGCACTT	TCGTATCCATCTGGCAGCTT
**α5 integrin**	CCGGGACACTAAGAAAACCA	CACGTCCTCCTCCTTCTGAG
**α6 integrin**	GGAGCCCCACAGTATTTTGA	TTCCATTTGCAGATCCATGA
**αv integrin**	CACCAGCAGTCAGAGATGGA	TGCCTTGCTGAATGAACTTG
**PECAM1**	GATTTCTCATGACGCCCAGT	ACGTCTTCAGTGGGGTTGTC
**NCAM1**	CTCGAAAGACGAGTCCAAGG	CTGGCTTCGTTTCTGTCTCC
**GAPDH**	GGTCGGAGTCAACGGATTTGGTCG	GCCAGCATCGCCCCACTTGA

*, Sequence obtained from Esnault et al [27]

### Western Blotting

Total lysates were obtained by incubating cells in RIPA lysis buffer (ThermoScientific) supplemented with 1% Protease Inhibitor Cocktail (Sigma-Aldrich-, St. Louis MO) for 1 h at 4 (C. The lysate was centrifuged at 14,000 x g for 20 min at 4 (C and a protein assay was performed on the supernatant using the BCA assay kit (ThermoScientific). The supernatant was mixed with NuPAGE LDS sample buffer (Invitrogen) containing DTT and heated at 70°C for 10 min. The samples were centrifuged at 13,000 x g for 2 min and loaded on 4–12% Bis-Tris SDS-NuPAGE gels (Invitrogen) in MES buffer (for galectin-1) and MOPS buffer for analyzing other proteins at 10 μg protein per lane. After electrophoresis the proteins were transferred to PVDF (BioRad, Hercules CA). The membrane was blocked for 1 h in 5% Blotto in TBST (Tris buffered saline plus 0.5%Tween 20). The blocked membrane was incubated overnight with primary antibody in 5% Blotto in TBST, then washed and incubated with secondary antibody labeled with horseradish peroxidase (ThermoScientific). After further washing, the membrane was incubated with chemiluminescent substrate (WesternBright Quantum, E&K Scientific, Santa Clara, CA) and then exposed to a Kodak imager (Kodak Imaging Systems, New Haven CT). Densitometry was performed using Kodak Carestream Molecular Imaging Softwareand normalized to GAPDH control.

### Zymography

Gelatin zymography was used to measure the activity of MMP2 and MMP9. 5μl of culture supernatant was added to 5μl of 2X Tis-glycine SDS sample buffer (Life Technologies, Grand Island, NY) and incubated for 10 min at room temperature. The samples were loaded onto a 10% zymogram gel supplemented with 1% gelatin (Life Technologies, Grand Island, NY). Electrophoresis, renaturing and development of the gels were performed according to the manufacturer's instructions (Life Technologies, Grand Island, NY). The developed gels were stained with Simply Safe blue stain (Life Technologies, Grand Island, NY). The gels were photographed and the proteolytic activity was determined by densitometric analysis using Image J software.

### Proliferation Assay

In order to correctly interpret the results of the invasion assay (see next paragraph) it was first necessary to test the effect of LiCl on cell proliferation. To test the effect of LiCl on cell proliferation equal numbers of trophoblasts were incubated in the presence either NaCl (control) or LiCl for 7 days after which DNA was quantified using a CyQuant Proliferation assay kit (Life Technologies, Grand Island NY) following the manufacturer’s instructions.

### Invasion Assay

An invasion assay was performed using the CytoSelect 24 well, 8μm invasion assay kit (Cell Biolabs Inc.,San Diego, CA). Briefly, trophoblast cells were cultured in the inserts and treated for 7 days in the presence of sodium chloride or lithium chloride. The inserts were removed from the medium and any cells on the lower surface were removed by using a sterile cotton swab. The inserts were placed in complete medium in the lower chamber and medium with no FBS was added to the upper chamber. 72h after incubation, invasion was assessed as described by the manufacturer.

### Coculture Experiments

For trophoblast/endothelial cell co-culture experiments, UtMVECS were cultured to confluence in 2-well LabTek permanox slides under 2% oxygen. Trophoblasts that had been treated for 7 days in the presence of lithium chloride or sodium chloride were trypsinized and resuspended in trophoblast medium. Cells were counted and 10,000 cells were added per well. Slides were cultured in trophoblast medium and photographed, fixed and stained at different time points.

### Statistical Analyses

All experiments were repeated at least 3 times. Statistical analysis was performed by two-way ANOVA with repeated measures for the time-course experiments and by Student’s *t test* for all other data using the Prism software program (GraphPad Inc., San Diego, CA). Data are expressed as means ± SEMs and differences were considered significant if *p* < 0.05.

## Results

### Characteristics of Isolated Cells

**Fig 1:**
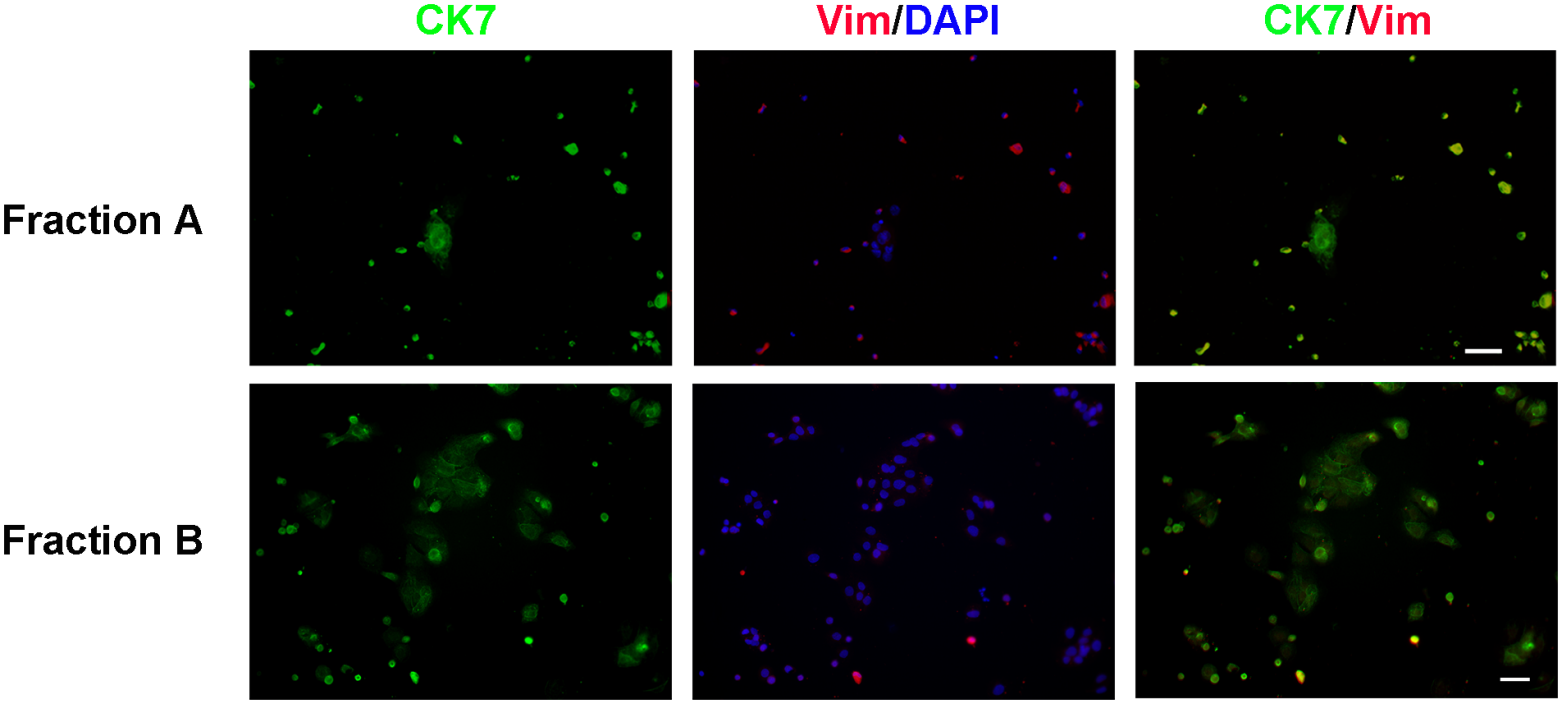
Characterization of trophoblasts isolated from early gestation rhesus monkey placenta. Trophoblasts were isolated as described in Methods. Fraction A represents the pool of tissue digests 1–3 and fraction B represents the pool of digests 4–8. The cells were cultured for 24 h and then fixed, permeabilized and stained using antibodies against cytokeratin 7 (green) and vimentin (red) as described in Methods. Nuclei were identified using DAPI (blue). The white bars represent 20 μm. The images are representative of cells isolated from 4 different placentas.

Fig 1 shows immunofluorescence analysis of cytokeratin 7 (CK7; trophoblast marker) and vimentin (Vim; mesenchyme marker) expression after culture of cells from fractions A and B (see Methods) on collagen-coated glass slides for 24 h. Cells obtained from Fraction A were small and rounded and were consistently contaminated with particulate debris. These cells adhered poorly and did not survive cryopreservation well. Cell yields were also very low. Most of the cells in Fraction A were positive for CK7 but many of these cells also co-expressed vimentin. This visual impression was confirmed by image analysis of the stained cells (Table 3) where it can be seen that 55.9% of the cells co-expressed both proteins. Less than 1% of the cells were CK7^-^Vim^+^.

**Table 3:** Characterization of isolated trophoblasts. Cells were cultured for 24h and then stained with antibodies against cytokeratin 7 (CK7) and vimentin (Vim) as described in Methods. Nuclei were stained with DAPI. Total nuclei, total CK7+ cells and total vimentin+ cells were counted. These values were used to derive the four parameters shown in the table as described in Methods. Values represent means±SEM for 4 experiments.

Population	Fraction A	Fraction B
	%	%
**CK7** ^**+**^ **Vim** ^**-**^	38.8±12.9	93.9±1.3
**CK7** ^**+**^ **Vim** ^**+**^	55.9±11.2	2.7±0.2
**CK7** ^**-**^ **Vim** ^**+**^	0.7±0.7	1.2±0.6
**CK7** ^**-**^ **Vim** ^**-**^	4.6±4.6	2.2±0.8

In contrast, cells from Fraction B adhered well and consistently survived cryopreservation with little loss of viability. The cultures consisted of some single cells but large irregular-shaped cell colonies predominated. Immunofluorescence analysis revealed very few or no vimentin^+^ cells and the majority of cells expressed CK7 only consistent with trophoblasts (Fig 1, lower panel). Image analysis of the stained cells (Table 3) showed that 93.9% of the cells in Fraction B were CK7^+^Vim^-^. No staining was found for the endothelial marker, Factor VIII, the uterine epithelial marker, glycodelin, or the hematopoietic precursor cell marker CD34 (See S3 and S4 Figs). Only cells from Fraction B were used for the present studies.

### Effect of Sodium Butyrate on Trophoblasts

**Fig 2:**
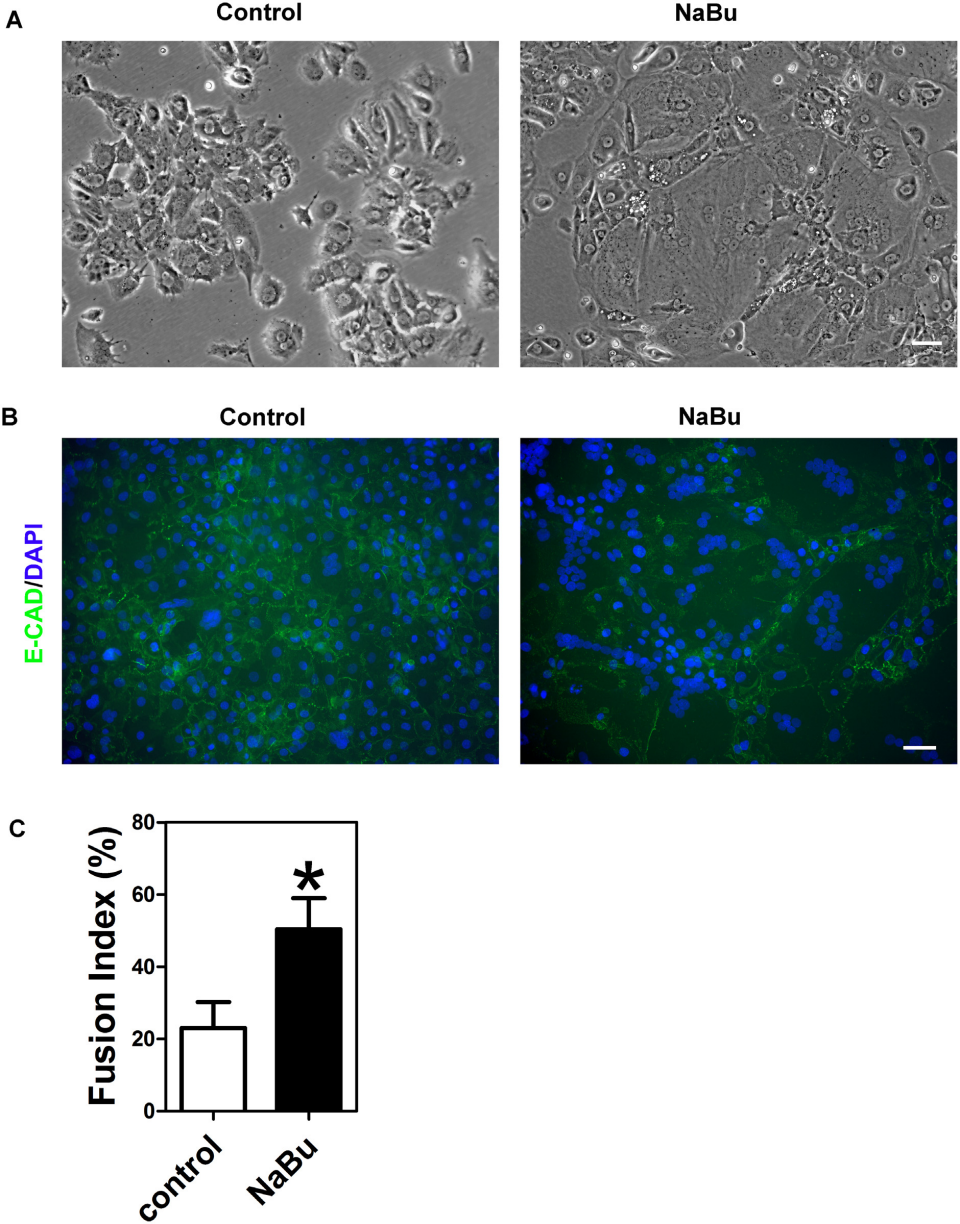
Effect of sodium butyrate on the formation of multinucleated syncytiotrophoblast. Trophoblasts were incubated in the presence or absence of sodium butyrate (0.5 mM) for 7 days. (A) Phase contrast images of live cells. (B) Merged immunofluorescence images of cells stained using an antibody against E-cadherin (green) and DAPI (blue). (C) Graph showing the fusion indices for cells cultured in the absence or presence of sodium butyrate. The fusion index was calculated as described in Methods. The results are means ± SEM from three experiments. The asterisk indicates the value is significantly different (p<0.05) from the control.

When trophoblasts were incubated with sodium butyrate the cell colonies became flatter and, unlike control cultures, it was difficult to distinguish individual cells (Fig 2A). When sodium butyrate-treated cells were stained with the cell junction marker E-cadherin, large numbers of multinucleated cells were seen whereas control cells mostly appeared to be mononucleated (Fig 2B). This impression was confirmed by calculating the cell fusion index which showed significantly greater numbers of multinucleated cells in the presence of sodium butyrate compared to the untreated controls (Fig 2C).

**Fig 3:**
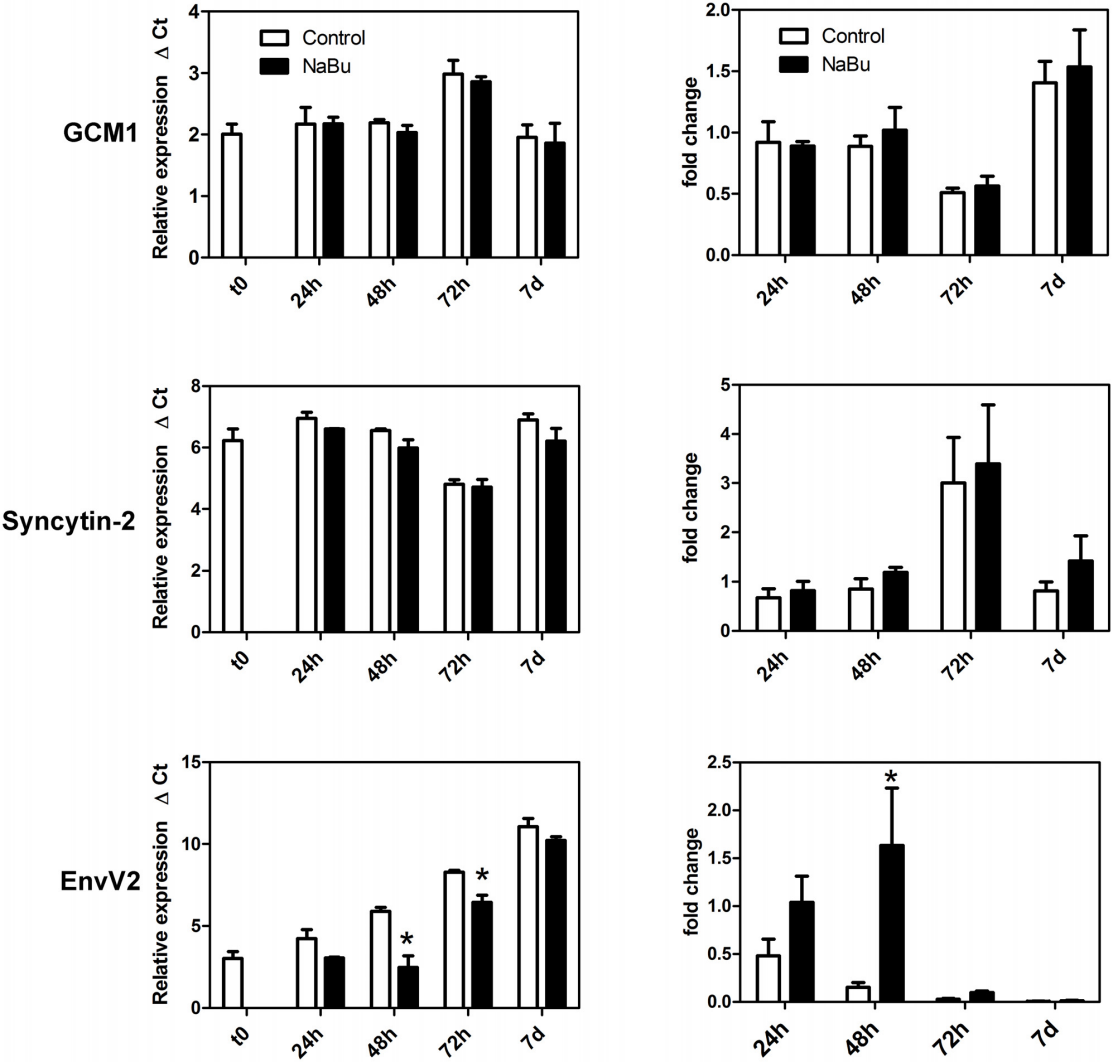
Effect of sodium butyrate on the expression of selected trophoblast genes. Trophoblasts were incubated in the presence or absence of sodium butyrate for different times as indicated on the graphs. At each time point gene expression was measured by qPCR as described in the Methods section. The graphs on the left show relative expression as mean ΔCt values ± SEM for three experiments and higher values represent lower expression. The asterisks indicate that the values are significantly different from the respective control values (p<0.05, ANOVA with Bonferoni post-test). The graphs on the right represent fold-change relative to t = 0.

The transcription factor GCM1 plays an important role in regulating the formation of multinucleated syncytiotrophoblast in the human by controlling the expression of two endogenous retroviral genes, syncytin-1 and syncytin-2 [28], which are key factors in the fusion process. We therefore sought to determine whether the sodium butyrate-induced formation of syncytiotrophoblast was accompanied by changes in the expression of these factors. The results in Fig 3 show that relative mRNA levels of GCM1 and syncytin-2 in butyrate-treated cells were not significantly different from untreated controls over the course of 7 days. However, mRNA for EnvV2, another endogenous retroviral gene expressed specifically in human and non-human primate placentas [27, 29], was significantly upregulated at 48h in the presence of sodium butyrate compared to the untreated control (Fig 3). Primate EnvV2 (but not human EnvV2) is fusogenic in cell transfection studies [27]. Syncytin-1 was not investigated because this gene is not expressed in rhesus monkeys [30].

**Fig 4:**
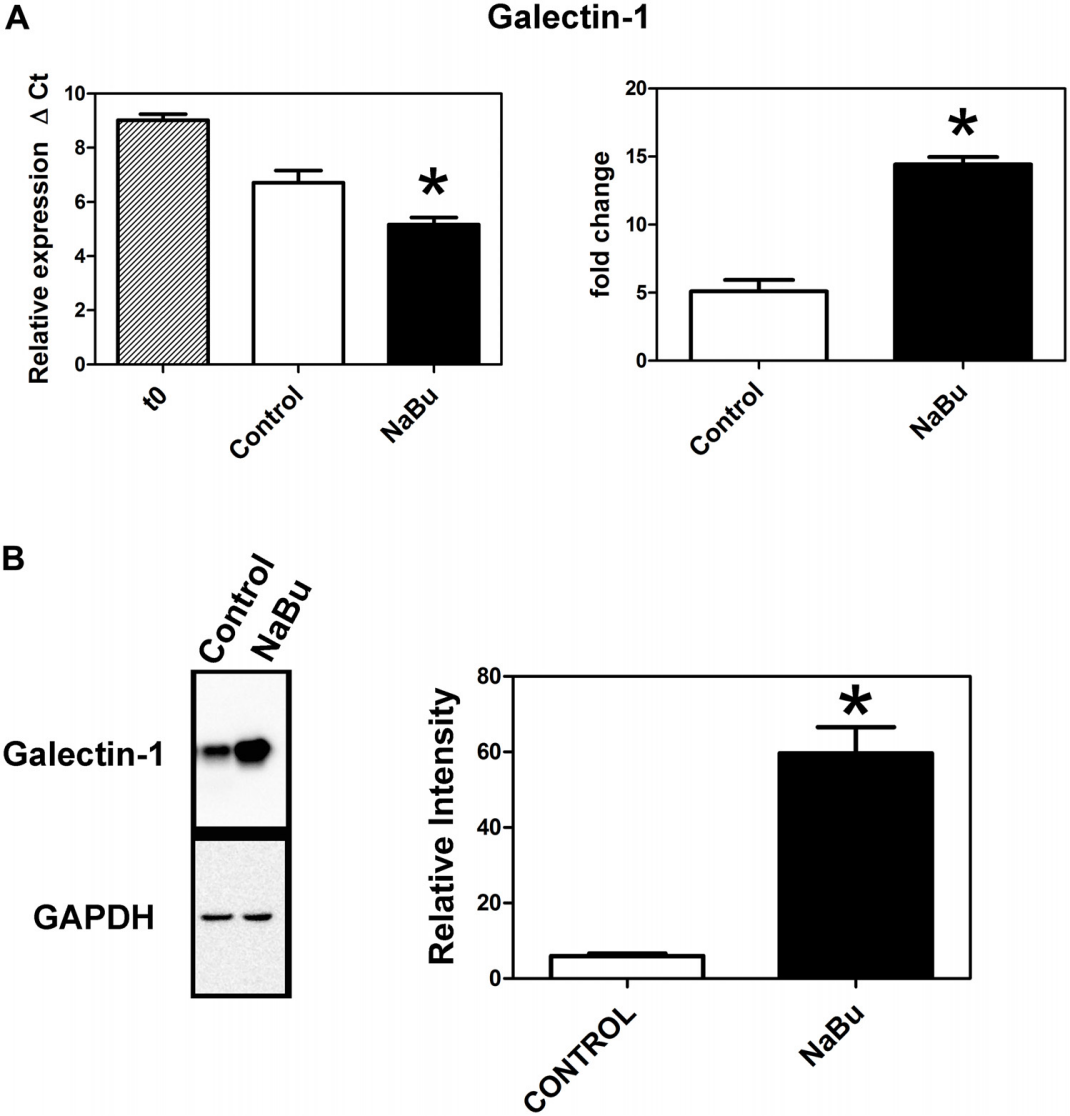
Effect of sodium butyrate on the expression of Galectin-1. Trophoblasts were incubated in the presence or absence of sodium butyrate for 7 days after which the expression of galectin-1 was measured by (A) qPCR and (B) Western blot as described in Methods. In A the graph to the left shows relative expression as mean ΔCt ± SEM for three experiments. The graph on the right show fold-change relative to t = 0. In B the graph to the right of the Western blot shows the results of densitometric quantitation (n = 3 in each case) and the asterisks indicate values that are significantly different (p<0.05) from the respective control values.

We also found (Fig 4A and 4B) that, compared to the control, sodium butyrate significantly increased the expression of galectin-1 at both the mRNA and protein levels. Galectin-1 may also play a role in the regulation of trophoblast fusion [31, 32].

### Does the Effect of Sodium Butyrate on Trophoblast Differentiation Result from Activation of the Wnt/β-Catenin Pathway?

**Fig 5:**
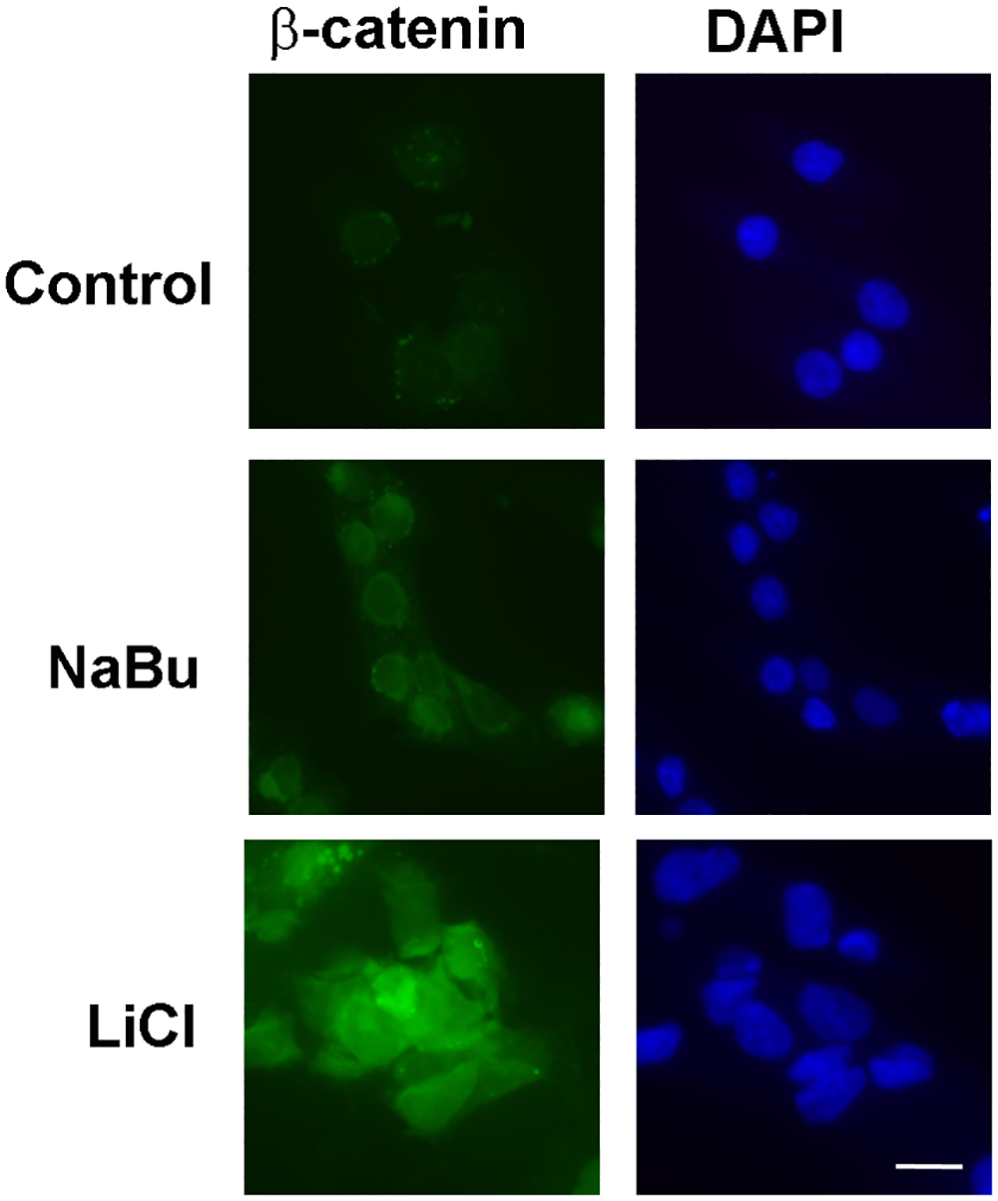
Effect of sodium butyrate and lithium chloride on nuclear accumulation of β-catenin in trophoblasts. The cells were incubated in the presence or absence of sodium butyrate, lithium chloride, or sodium chloride (control) 48h. The cells were then fixed and stained using an antibody against β-catenin (green) and DAPI (DAPI). The white bar represents 10 μm.

Sodium butyrate is known to activate the Wnt/β-catenin pathway [15, 33] and consistent with this we found that exposure of trophoblasts to sodium butyrate caused increased accumulation of β-catenin in nuclei compared to the control (Fig 5).

**Fig 6:**
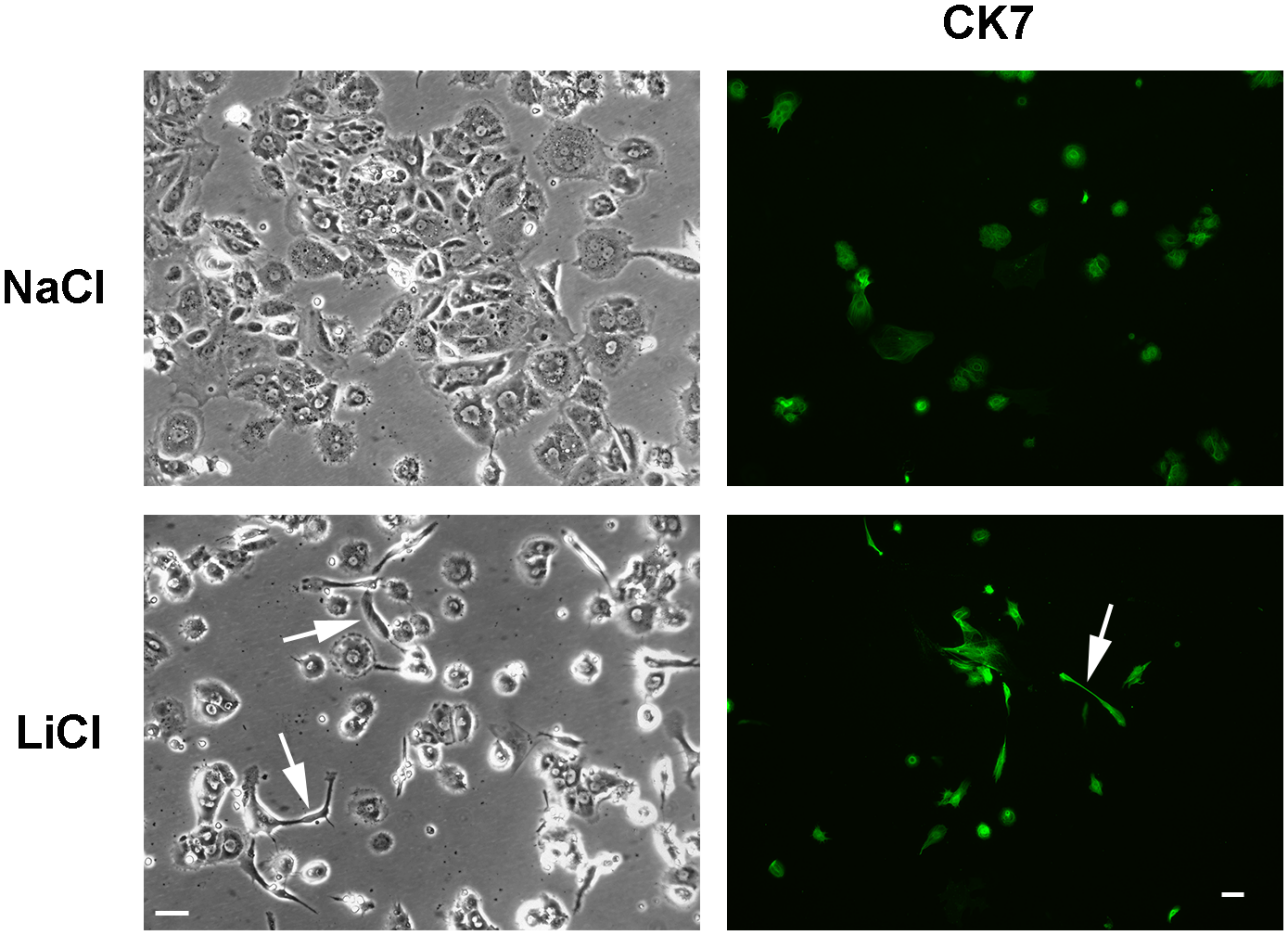
Effect of lithium chloride on trophoblasts. The cells were incubated in the presence of 20 mM lithium chloride or 20 mM sodium chloride (control) for 7 days and then stained using an antibody against CK7 as described in Methods. The white bar represents 20 μm. The arrows point to spindle-shaped cells in the lithium chloride-treated cultures.

To further explore the involvement of the Wnt pathway, trophoblasts were incubated in the presence of the GSK3 inhibitor LiCl. The protein kinase GSK3 plays an important role in the Wnt/β-catenin pathway and lithium chloride-mediated inhibition of GSK3 can mimic the effects of Wnt signaling [34, 35]. As expected, exposure to LiCl caused increased nuclear accumulation β-catenin compared to controls (Fig 5, lower panel). However, in contrast to sodium butyrate, LiCl failed to induce the formation of multinucleated syncytiotrophoblast. Instead, cells incubated with LiCl remained largely mononucleated and many spindle-shaped cells were observed (Fig 6). No spindle-shaped cells were observed in control cultures incubated in the presence of NaCl (Fig 6) or in the presence of butyrate (see Fig 2).

### Effects of LiCl on Extravillous Trophoblast Differentiation and Interaction with Endothelial Cells

**Fig 7:**
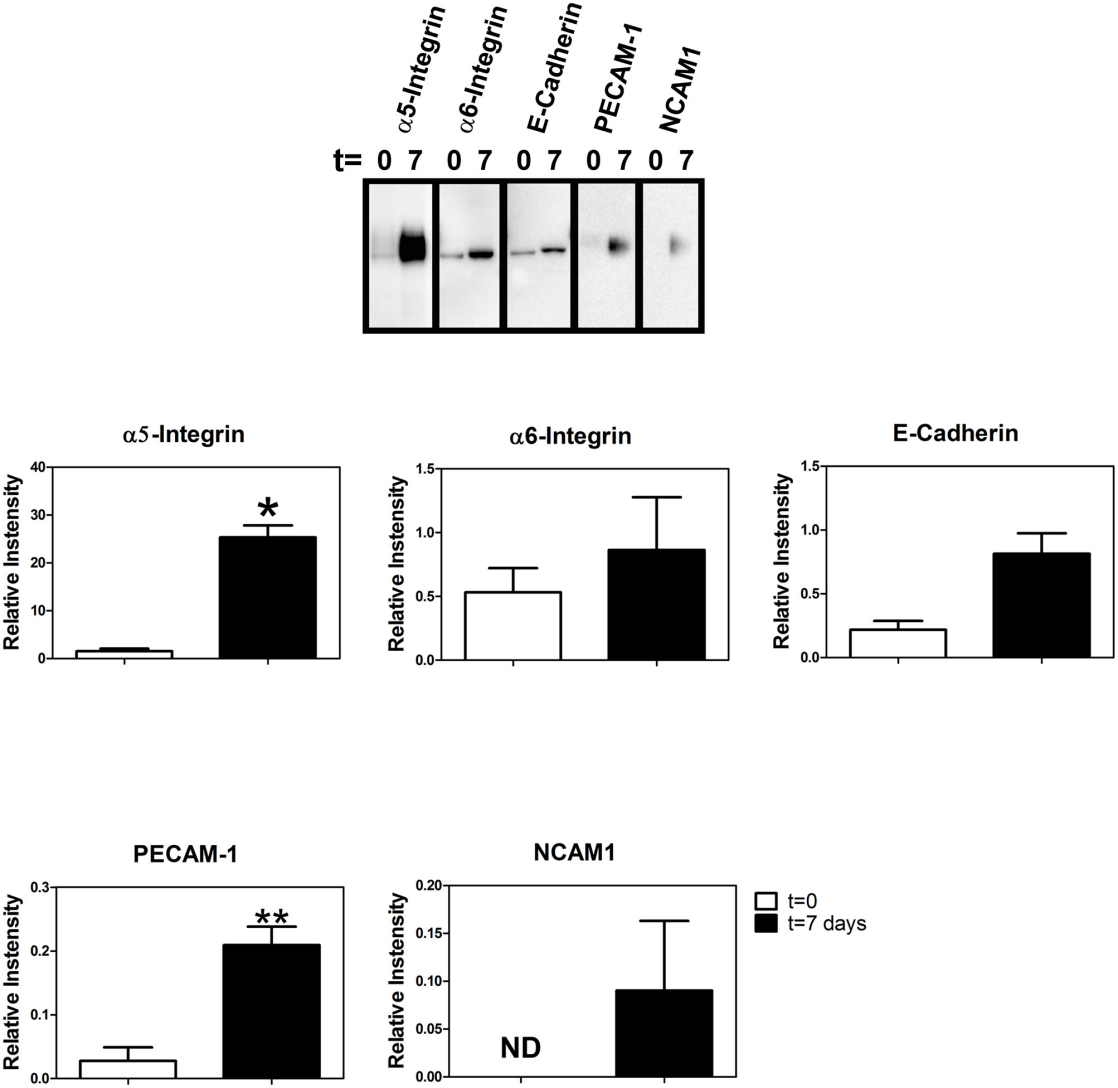
Expression of extravillous trophoblast marker proteins. Cells were cultured for 7 days (in the absence of sodium butyrate or lithium chloride) after which the expression of the selected adhesion molecules was detected by Western blotting as described in Methods. The graphs below the Western blot show densitometric quantitation of protein bands and values are shown as means ± SEM (n = 3). The asterisks indicate values that are significantly different (p<0.05) from the respective controls. ND, not detected.

**Fig 8:**
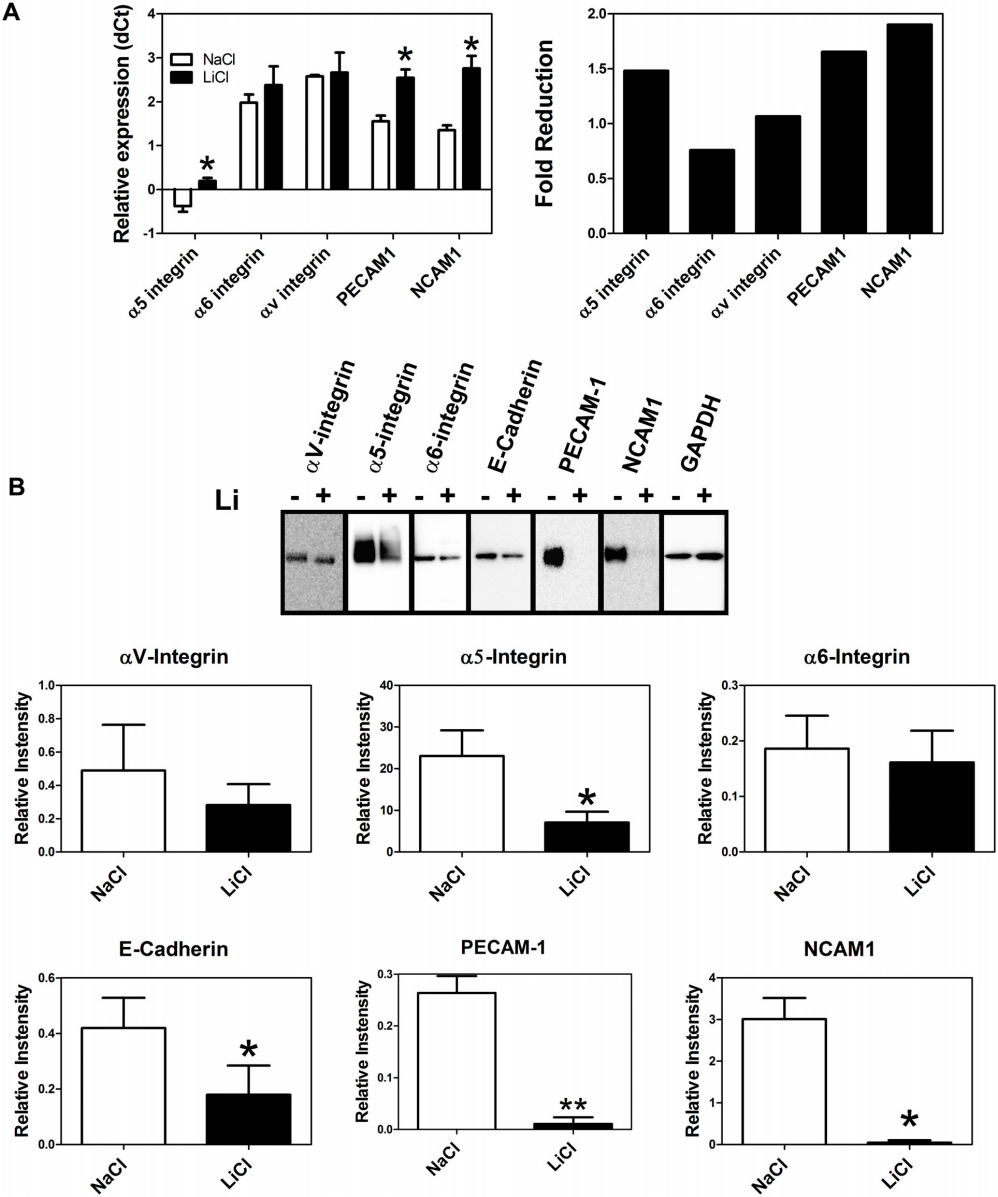
Effect of lithium chloride on trophoblast adhesion molecule expression. Trophoblasts were incubated in the presence of lithium chloride or sodium chloride (control) for 7 days after which the expression of the selected adhesion molecules was assessed (A) by qPCR and (B) by Western blot as described in Methods. In A the y axes show ΔCt values and so higher values represent lower expression. Note that values for fold- change are less than 1 (with respect to NaCl control) indicating reduced expression. The asterisks indicate that the values are significantly different from the respective control values (p<0.05, n = 3). The graphs in B show densitometric quantitation of protein bands and values are shown as means ± SEM (n = 3). The asterisks indicate values that are significantly different (p<0.05) from the respective controls.

**Fig 9:**
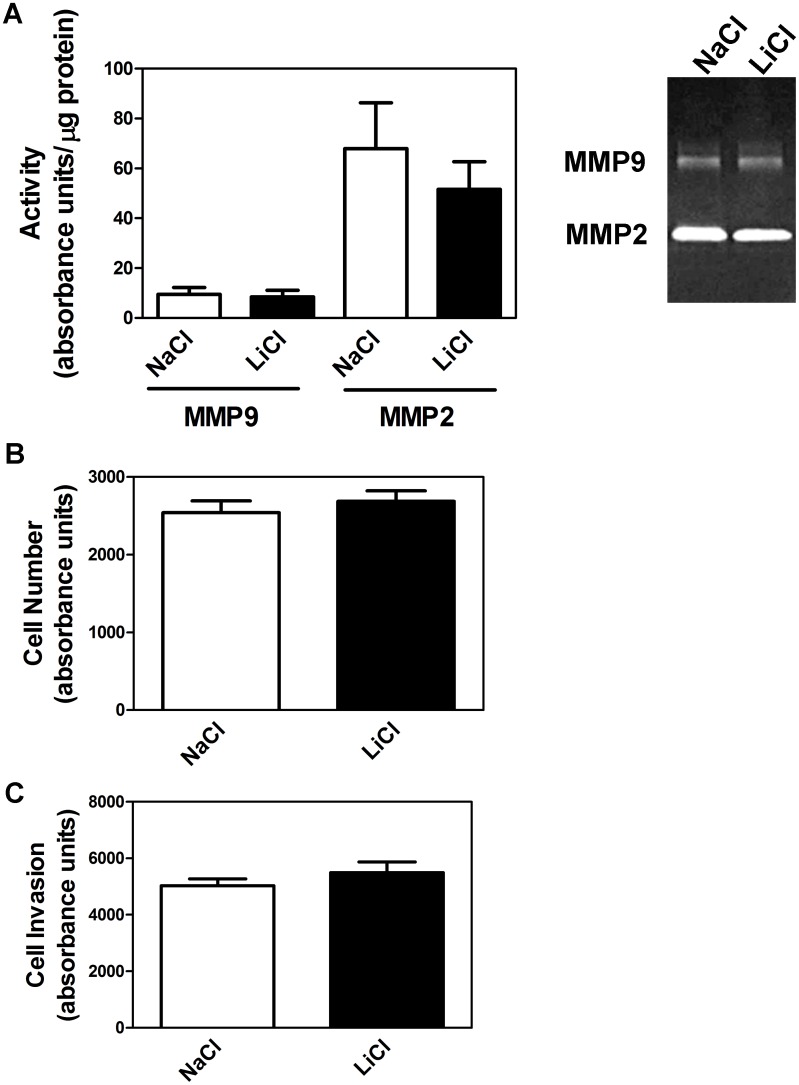
Effect of lithium chloride on metalloproteinase activity and cell invasion. (A) Cells were incubated in the presence of lithium chloride or sodium chloride (control) for 7 days after which MMP9 and MMP2 activities were measured by zymography as described in Methods. (B) To test for effects of lithium chloride on proliferation, equal numbers of trophoblasts were incubated in the presence of lithium chloride or sodium chloride for 7 days after which cell numbers were measured as described in Methods. (C) To assess invasive activity, trophoblasts were cultured in chamber inserts in the presence of lithium chloride or sodium chloride for 7 days after which invasion to the lower chamber was measured as described in Methods. Results are means ± SEM (n = 4).

When trophoblasts were incubated for 7 days in the absence of LiCl (or butyrate) we found significantly increased expression of α5-integrin and PECAM-1 consistent with extravillous trophoblast formation (Fig 7). However, when trophoblasts were incubated in the presence of LiCl, the expression of α5-integrin, PECAM-1, and NCAM1 was significantly reduced at the mRNA level (Fig 8A) and α5-integrin, E-cadherin, PECAM-1, and NCAM1 expression was significantly reduced at the protein level (Fig 8B). Although trophoblasts secreted MMP2 and MMP9 (again consistent with extravillous trophoblasts), lithium chloride had no effect on this secretory activity (Fig 9A). In order to further examine the effect of lithium chloride on trophoblast invasive potential, invasive activity was measured using in vitro invasion chambers. We first confirmed that LiCl had no effect on cell proliferation since this could have compromised interpretation of the invasion assay. As can be seen from Fig 9B there was no significant difference in cell numbers between cells incubated in the presence of LiCl or NaCl (control). No significant effect of lithium chloride on invasion was observed (Fig 9C).

**Fig 10:**
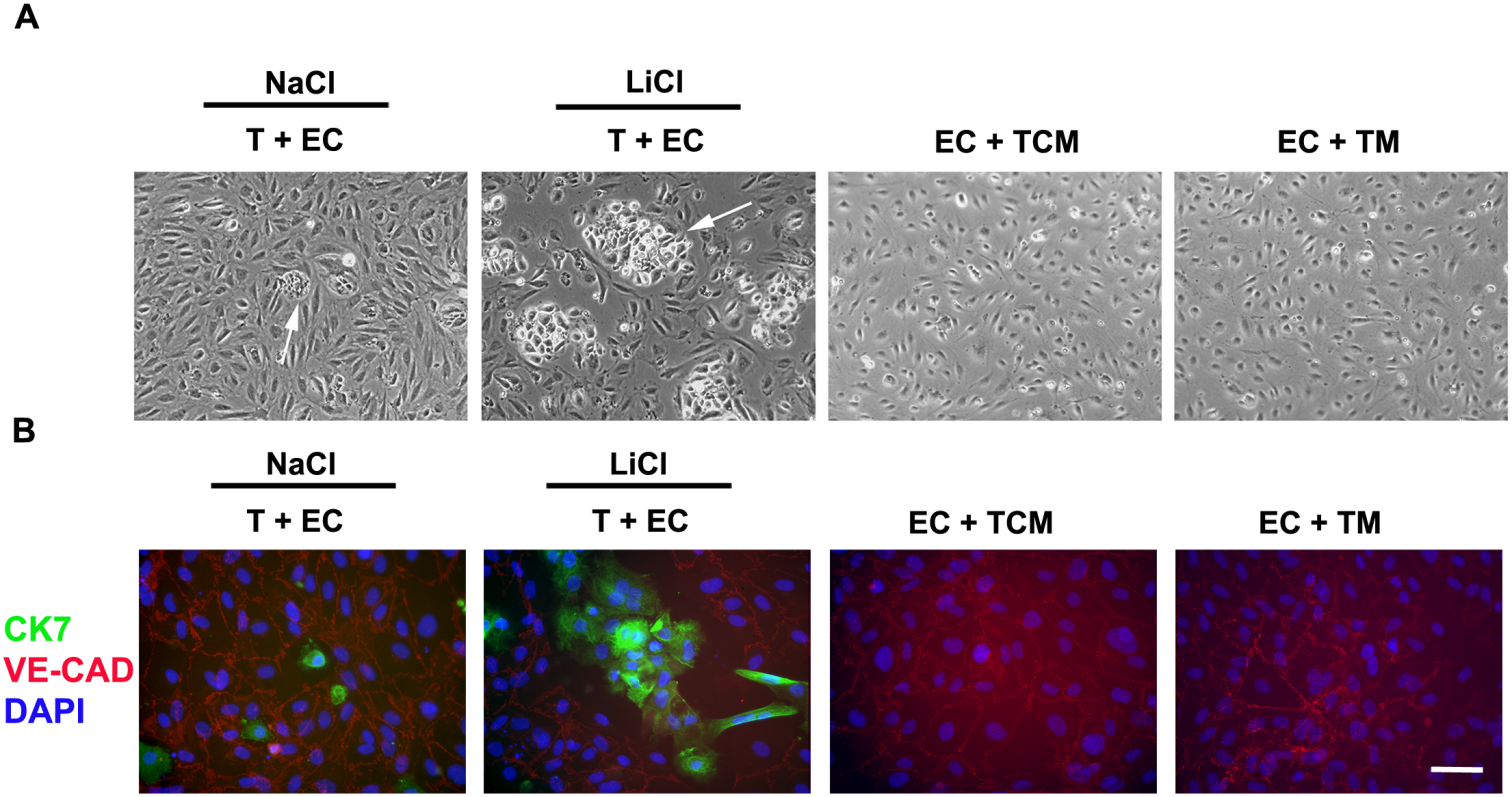
Effect of Lithium chloride on the interaction of trophoblasts with endothelial cells. Trophoblasts were incubated in the presence of lithium chloride or sodium chloride (control) for 7 days as described in methods. The cells were then trypsinized and added on top of confluent monolayers of uterine microvascular endothelial cells. The cells were cocultured for 48 h. Other endothelial monolayers were incubated with trophoblast-conditioned medium (TCM) or trophoblast medium (TM) (A) Phase-contrast image of live cocultures. The arrows indicate trophoblast colonies. Note the large colonies seen in the presence of lithium chloride. (B) Immunofluorescence staining of cocultures and endothelial monolayers using antibodies against CK7 (green), VE-cadherin (red) and DAPI (blue). T, trophoblasts; EC, endothelial cells. The white horizontal bar represents 20μm.

Reminiscent of the results reported here for PECAM-1, immunohistochemical studies in the rhesus monkey show that extravillous trophoblasts that have invaded uterine spiral arteries (endovascular trophoblasts) show reduced expression of PECAM-1 [36]. This suggested that LiCl could be inducing or increasing the formation of endovascular trophoblasts under our *in vitro* conditions. We therefore compared the behavior of LiCl-treated trophoblasts and control trophoblasts when added and cocultured on top of adherent endothelial cells. When trophoblasts which had been incubated in the presence of LiCl for 7 days were added to confluent cultures of endothelial cells, large rounded colonies of trophoblasts could be distinguished from surrounding endothelial cells by their distinctive refractive properties (Fig 10A). In contrast, trophoblasts preincubated with NaCl formed only small round colonies when plated on top of confluent endothelial cell monolayers (Fig 10A).

When the cocultures that contained LiCl-treated trophoblasts were stained using antibodies against CK7 (trophoblast marker) and VE-cadherin (endothelial cell marker), the trophoblast colonies appeared to be in “gaps” devoid of underlying VE-cadherin staining but closely abutted adjacent endothelial cells (Fig 10B). Endothelial cells incubated in the presence of trophoblast-conditioned medium or trophoblast medium remained confluent with no gaps (Fig 10B).

### Effect of the GSK3 Inhibitor CHIR99021 and TNFα on Trophoblast Differentiation

**Fig 11:**
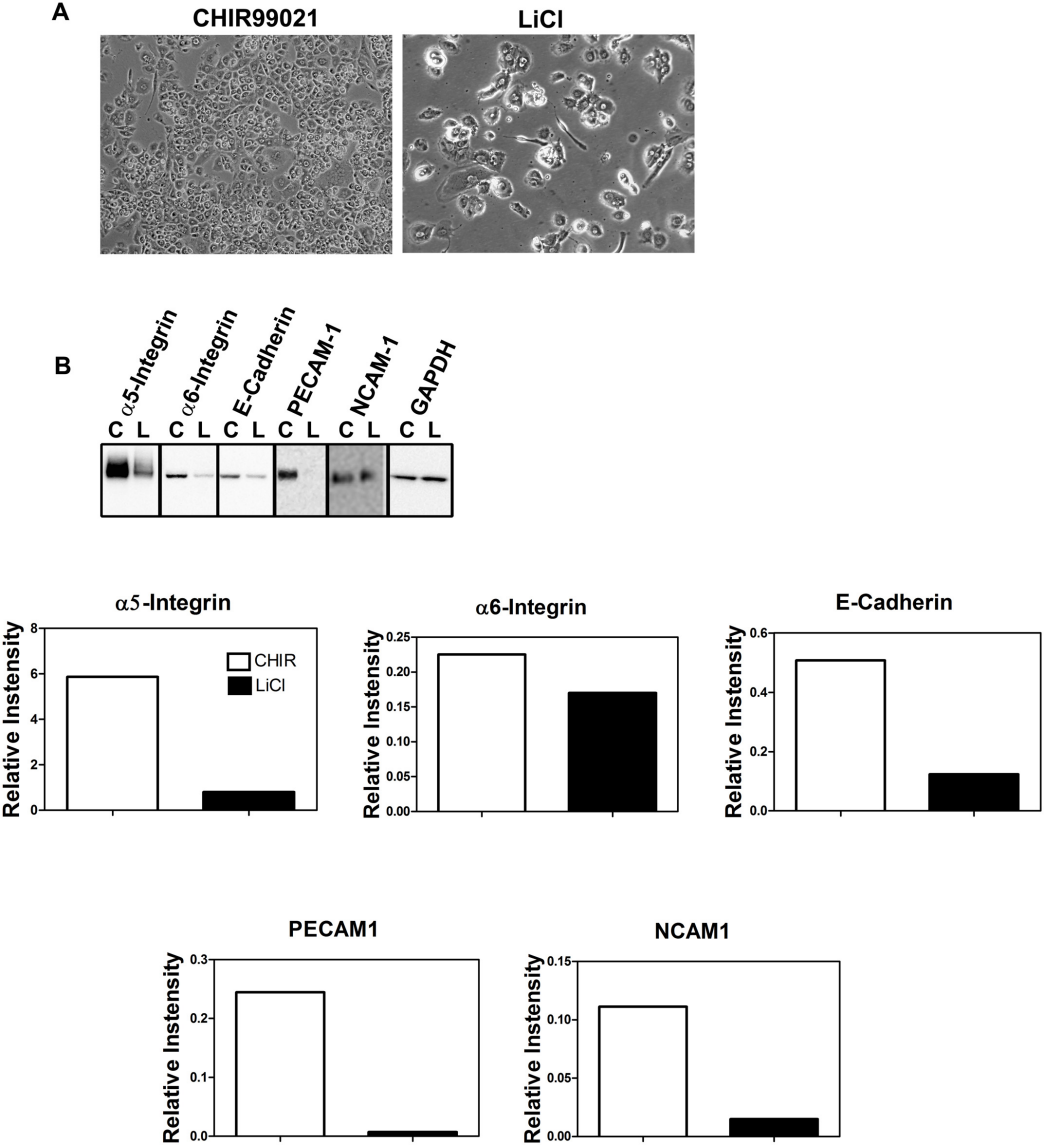
Comparison of the effects of CHIR99021 and lithium chloride on trophoblast adhesion molecule expression. Trophoblasts were incubated in the presence of 3 μM CHIR99021 or 20 mM lithium chloride for 7 days. (A) Phase contrast image of cells at 7 days. Note the absence of spindle-shaped cells in the presence of CHIR99021 and their presence when trophoblasts were incubated with lithium chloride. (B) Expression of adhesion molecules was assessed by Western blot as described in Methods. The graphs below the Western blot show densitometric quantitation of protein bands (n = 2). C, CHIR99021; L, lithium chloride.

Next we incubated trophoblasts with CHIR99021, a more highly specific inhibitor of GSK3. Unlike LiCl, this compound did not induce the appearance of spindle-shaped cells (Fig 11A) nor did it have the same effect as LiCl on the expression of trophoblast adhesion molecules (Fig 11B). This suggested that the effects of LiCl might be mediated via a non-GSK3 pathway.

**Fig 12:**
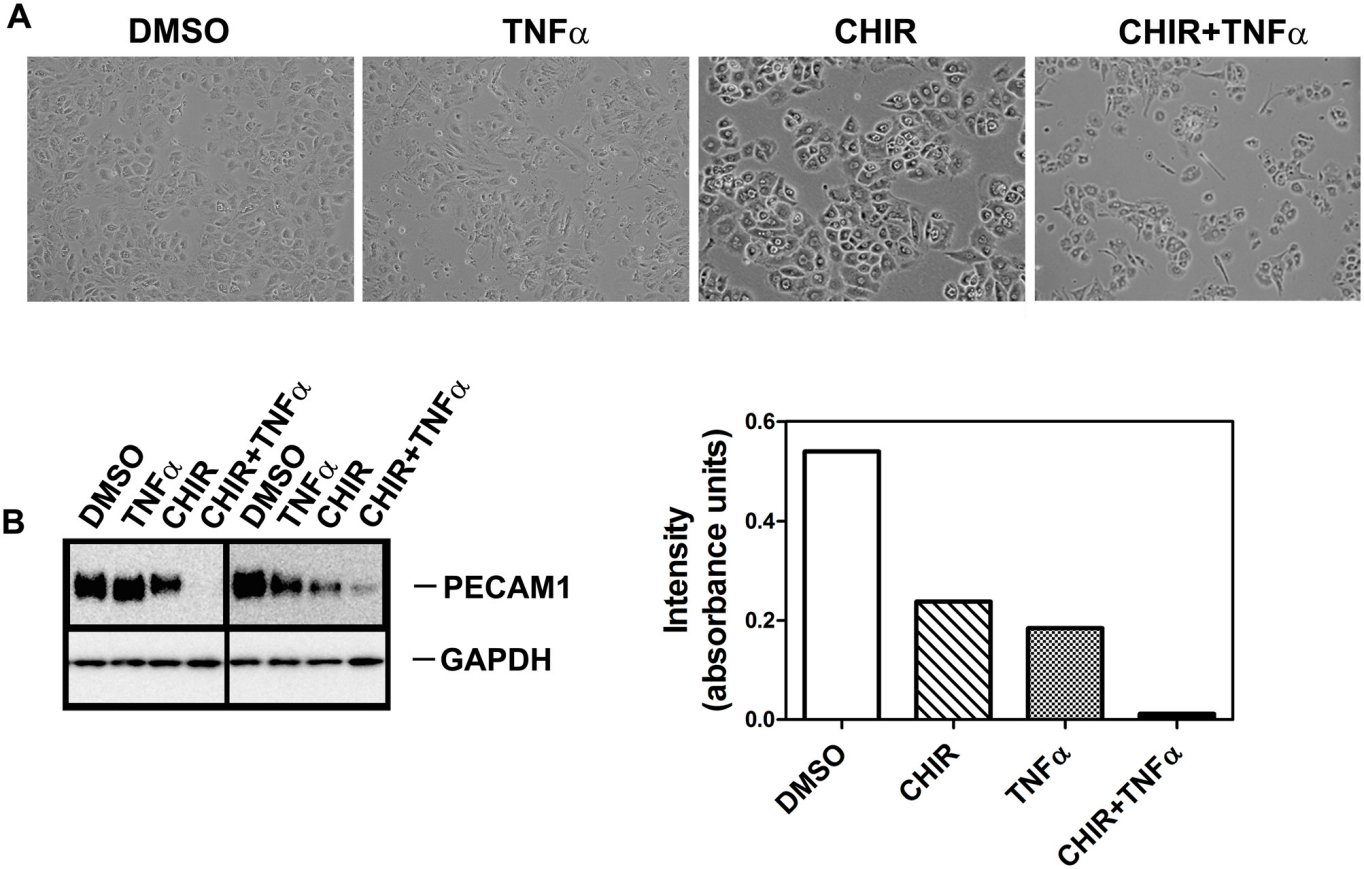
Effect of TNFα (10 ng/ml) on trophoblast PECAM1 expression in the presence of 3 μM CHIR99021. Trophoblasts were incubated in the presence of the indicated compounds for 3 days. (A) Phase-contrast images of live cells. (B) Analysis of PECAM1 expression by Western blot as described in Methods. The graph shows densitometric quantitation of the PECAM1 bands (n = 2). Final concentration of DMSO (vehicle control) was 0.03%.

TNFα is known to inhibit villous trophoblast differentiation [37] as well as trophoblast invasion [38, 39] and also reduces PECAM-1 expression in endothelial cells [40, 41]. We wondered whether the different effects of LiCl and CHIR99021 might be related to their differential effects on TNFα expression. LiCl has been reported to increase TNFα expression [42] whereas CHIR99021 is reported to reduce expression of pro-inflammatory cytokines including TNFα [43]. When trophoblasts were incubated with TNFα, numerous spindle-shaped cells appeared in contrast to cells incubated in the presence of CHIR99021 (Fig 12A). Cells incubated in the presence of both CHIR99021 and TNFα showed the appearance of spindle-shaped cells. Also, cells incubated in the presence of TNFα or CHIR9901 showed a reduction in expression of PECAM-1 (Fig 12B). Cells incubated in the presence of both CHIR99021 and TNFα showed a greater reduction in PECAM-1 expression than in the presence of either factor alone (Fig 12B).

## Discussion

Trophoblast differentiation is a complex and incompletely understood process that is essential for normal placental development and successful pregnancy outcome. In primates, two main pathways have been identified. The villous pathway results in the formation of multinucleated syncytiotrophoblast whereas the extravillous pathway results in the appearance of invasive trophoblasts (extravillous trophoblasts) [44]. Cells that follow the extravillous pathway can become interstitial extravillous trophoblasts residing in the decidua or endovascular trophoblasts that invade and migrate along uterine spiral arteries and remodel the vessel walls. How these cell fate decisions are orchestrated is not known. In the present paper we have shown that sodium butyrate and lithium chloride have different effects on these pathways although both compounds caused nuclear accumulation of β-catenin.

Placentation in the rhesus monkey shows many similarities to that in the human [45] and we have previously used this experimental system to investigate trophoblast migration [46–49]. In the experiments reported here we used early gestation rhesus monkey trophoblasts as a model to investigate the effects of the epigenetic modifier sodium butyrate on trophoblast differentiation. The experiments were conducted under 2% oxygen in order to mimic the environment encountered by trophoblasts during the early first trimester [19, 20]. Exposure of rhesus monkey trophoblasts to sodium butyrate consistently caused the appearance of increased numbers of multinucleated cells. These observations are consistent with the formation of syncytiotrophoblast. Additional support for this conclusion was provided by the finding that sodium butyrate increased the expression of EnvV2 (at the mRNA level) and galectin-1 (at the mRNA and protein levels), which are thought to be involved in trophoblast cell fusion [27, 31, 32]. Like the more frequently studied syncytins, EnvV2 is an endogenous retroviral-derived gene that is expressed by the non-human primate and human placenta [27, 50]. However, while the primate EnvV2 protein has fusogenic activity, the human protein does not [27, 50]. The increased expression of EnvV2 along with increased formation of multinucleated cells in the presence of sodium butyrate is consistent with a role for EnvV2 in rhesus monkey trophoblast villous differentiation. Galectin-1 has been shown to be involved in human trophoblast cell fusion [31, 32] and so the increased expression of galectin-1 during butyrate-induced syncytiotrophoblast formation in the rhesus monkey is consistent with this role. Our findings are also consistent with earlier studies which showed that galectin-1 expression was increased in human intestinal epithelial cells by sodium butyrate [51, 52].

Although the rhesus monkey trophoblasts expressed syncytin-2 and GCM1, two other genes with major roles in syncytiotrophoblast formation in the human, we found no evidence that sodium butyrate altered the expression of these genes at the transcriptional level in rhesus trophoblasts. However, this does not rule out changes at the translational level nor does it rule out their involvement in syncytiotrophoblast formation. We were unable to assess translational changes due to lack of antibodies reactive with rhesus monkey GCM1, syncytin-2, and EnvV2. Although GCM1 plays a major role in regulating the expression of syncytins [28] it is not known whether it regulates EnvV2 expression. It should be noted that human trophoblasts also express syncytin-1 which plays a role in fusion [53] but this gene is inactive in non-human primates [30].

While the effect of sodium butyrate reported here provides a useful tool for manipulating syncytiotrophoblast formation in vitro, its mechanism of action remains elusive. Sodium butyrate has a wide range of effects on cells [8–13]. Many of these effects are likely the result of inhibition of histone deacetylase activity [13] but other mechanisms may also be involved [14]. In the present studies we found that sodium butyrate increased the nuclear accumulation of β-catenin consistent with its known ability to activate Wnt signaling [15, 33]. However, the likelihood that the effects of butyrate on syncytiotrophoblast formation are solely mediated by activation of canonical Wnt signaling is unlikely because syncytiotrophoblast formation was not induced when cells were incubated with the Wnt activator LiCl. Lithium chloride is known to mimic the effects of Wnt signaling through inhibition of GSK3 activity and stabilization of β-catenin [34, 35]. We also found that recombinant Wnt had no effect on syncytiotrophoblast formation despite the fact that the cells expressed frizzled-5 and endogenous Wnt3A and Wnt7A (see S8 Fig). There is good evidence that Wnt signaling plays a role in murine placental development and in the formation of trophoblastic giant cells and Wnt ligands and frizzled receptors are expressed by human trophoblasts [4, 5]. The Wnt pathway has been suggested to play a role in human trophoblast invasion [4] and cell fusion [6]. The results presented here are consistent with the involvement of Wnt activation in both differentiation pathways but suggest that Wnt activation *per se* does not determine specific cell fate decisions.

When cultured in the absence of LiCl (or butyrate) rhesus monkey trophoblasts showed significantly increased expression of α5-integrin and PECAM-1 over 7 days in culture consistent with acquisition of an extravillous phenotype. Surprisingly, although lithium chloride failed to induce syncytiotrophoblast formation, as described above, it significantly reduced the expression of α5-integrin, PECAM1, E-cadherin, and NCAM1 compared to control cells. The appearance of spindle-shaped cells was also observed in the presence of LiCl. Although trophoblasts also expressed αV-integrin (another protein associated with interstitial and endovascular extravillous trophoblasts), expression was not significantly altered by exposure to lithium chloride. A large number of immunohistochemical studies of first trimester human placental tissues have demonstrated increased expression of α5-integrin as extravillous trophoblasts change from the proliferative phase to the more invasive stage [54–57]. Also, *in vitro* studies support the idea that α5-integrin is directly involved in regulating trophoblast invasion [58]. Invasive extravillous trophoblasts also acquire expression of NCAM1 and this is also expressed by endovascular trophoblasts in the human and the rhesus monkey [59, 60]. In the rhesus monkey, although NCAM1 is expressed by endovascular trophoblasts within the lumen of uterine spiral arteries it is not expressed by intramural trophoblasts distal to the lumen [60]. Again in the rhesus monkey, PECAM1 expression was observed in extravillous trophoblasts including those within the lumen of uterine spiral arteries but expression was reduced in intramural trophoblasts more distant from the lumen[36]. The expression of PECAM1 by human extravillous trophoblasts is controversial [61–63]. Increased expression of E-cadherin during human extravillous trophoblast differentiation has been reported but, again, the findings are controversial [62, 64]. Taken together with these published findings, our results are consistent with the idea that LiCl induces differentiation of an endovascular trophoblast phenotype and we speculate that this may be represented by the spindle-shaped cells that were observed under these conditions. Further studies will be required to test this hypothesis.

The idea that lithium chloride induced the formation an endovascular trophoblast phenotype is supported by the observation that lithium chloride-treated trophoblasts behaved differently from control trophoblasts when added to confluent endothelial cell monolayers. The lithium-treated trophoblasts formed large rounded colonies that attached to the substrate in intercellular gaps that formed within the endothelial monolayers in regions of trophoblast-endothelial cell contact. These gaps were devoid of endothelial cells since staining for VE-cadherin was negative in these areas. The gaps in the endothelial cell monolayers only appeared in the presence of trophoblasts and were not observed when endothelial cells were exposed to trophoblast conditioned-medium. Although the latter observation suggests that the endothelial gaps were not caused by factors secreted by the trophoblasts, it is possible that close contact of trophoblasts and endothelial cells creates a localized microenvironment in which high concentrations of secreted factors accumulate. Previous *in vitro* coculture studies have also shown that trophoblasts attach to and erode/displace underlying endothelial cells [65–69]. It has also been shown that trophoblast-derived factors alter the permeability of endothelial cell monolayers [69]. An alternative explanation for the lithium chloride-induced changes in trophoblast gene expression may simply be that the compound prevents acquisition of an extravillous phenotype and maintains the cells in a progenitor state.

The mechanism by which lithium chloride induced these changes in trophoblast cells does not seem to be directly related to its ability to inhibit GSK3 because of the results obtained using another GSK3 inhibitor, CHIR99021. This compound is highly specific for GSK3 and, unlike LiCl, failed to alter the acquisition of extravillous trophoblast adhesion molecules and also failed to induce the formation of spindle-shaped cells. Together, these results suggest that the effects of LiCl are not mediated through direct inhibition of GSK3 and not directly through activation of canonical Wnt signaling. Further experiments will be required to better understand these observations.

While recognizing that it is difficult to uncover the mechanisms of action of sodium butyrate and lithium chloride on trophoblast differentiation, a possible explanation of their differential effects was suggested by the experiments using TNFα. We found that incubation of trophoblasts with TNFα, like LiCl, reduced the expression of PECAM1 and induced the formation of spindle-shaped cells. We therefore speculate that the effects of LiCl on trophoblasts might be related to increased TNFα production. LiCl has been reported to increase TNFα expression in neutrophils [42]. The inhibitor CHIR99021 is reported to reduce expression of pro-inflammatory cytokines including TNFα [43] and we speculate that this may partially explain why its effects on trophoblasts were different from those of LiCl. It does not however explain the additive effect of TNFα and CHIR99021 on inhibition of PECAM1 expression. It should also be noted that sodium butyrate is reported to inhibit TNFα synthesis in various systems [70, 71] and that TNFα has been found to inhibit trophoblast invasion and α5-integrin expression [38, 39] as well as inhibiting syncytiotrophoblast formation [37].

In summary, the results presented here show that sodium butyrate and lithium chloride have differential effects on the differentiation of rhesus monkey trophoblasts although both induce nuclear accumulation of β-catenin. The ability of sodium butyrate and lithium chloride to induce syncytiotrophoblast formation and endovascular trophoblast formation, respectively, provides useful tools for manipulating trophoblast differentiation *in vitro* and may also assist in uncovering how trophoblast cell fate decisions are regulated during early pregnancy. Finally, it is suggested that future studies address the pathway-selective effects of butyrate and LiCl and that these may be related to their differential effects on TNFα production.

## Supporting Information

S1 Table Data used to calculate the fusion index. Cells were incubated with NaCl (control) or sodium butyrate (NaBu) and then fixed and stained for E-cadherin and DAPI as described in Methods. Plac1, Plac2, and Plac3 represent results from three different placentas. Three replicates were analyzed within each experiment.
(XLSX)

S2 Table QPCR data used to calculate relative expression of GCM1, Syn-2 and EnvV2 mRNA. (XLSX)

S3 Table Data used to calculate the relative expression of galectin-1 mRNA and protein. (XLSX)

S4 Table Data used for densitometric quantitation of trophoblast marker proteins shown in Fig 7. (XLSX)

S5 Table Data used for quantitation of adhesion molecule expression shown in Fig 8. (XLSX)

S6 Table Data used for assessment of trophoblast proliferation, MMP secretion, and invasion. Assessment of trophoblast proliferation, MMP secretion and invasion was carried out as described in Methods. Results from 4 different experiments are shown.
(XLSX)

S7 Table Comparison of the effects of CHIR99021 and lithium chloride on trophoblast adhesion molecule expression. (XLSX)

S8 Table Effect of TNFα on trophoblast PECAM1 expression in the presence of CHIR99021. (XLSX)

S1 Fig Characterization of cells in fraction A. Cells were stained for cytokeratin 7 (CK7), vimentin and DAPI as described in Methods and Fig 1. Each row represents cells from a different placenta.
(TIF)

S2 Fig Characterization of cells in fraction B. Cells were stained for cytokeratin 7 (CK7), vimentin and DAPI as described in Methods and Fig 1. Each row represents cells from a different placenta.
(TIF)

S3 Fig Absence of positive immunostaining for Factor VIII in trophoblasts. Cells were fixed and stained for immunofluorescence detection of Factor VIII as described in Methods. The antibody against Factor VIII was obtained from Santa Cruz Biotechnologies (sc27647). Nuclei were stained using DAPI. Each row shows factor VIII staining and the respective DAPI stain of the same field of view. Each row represents samples from different placentas. As a control, cells were incubated with non-immune goat Ig.
(TIF)

S4 Fig Absence of positive immunostaining for CD34 and glycodelin in trophoblasts. Cells were fixed and stained for immunofluorescence detection of CD34 and glycodelin as described in Methods. Antibody against CD34 was from Abcam (ab30375). Antibody against glycodelin was from Santa Cruz Biotechnologies (sc57511). Nuclei were detected using DAPI. Images show staining from cells from two different placentas in each case. Each row shows DAPI staining and the respective antibody stain. Note that the level of staining was similar to the background staining seen using control mouse Ig in place of the primary antibodies.
(TIF)

S5 Fig Images used to calculate the Fusion Index (control). Control cells were incubated in the absence of NaBu as described in Methods and Fig 2 and then stained with anti-cadherin antibody (green) and DAPI (blue). Each row shows triplicate samples from a different placenta (Plac 1–3).
(TIF)

S6 Fig Images used to calculate the fusion index (sodium butyrate). Control cells were incubated with NaBu as described in Methods and Fig 2 and then stained with anti-cadherin antibody (green) and DAPI (blue). Each row shows triplicate samples from a different placenta (Plac 1–3).
**levels.** QPCR data and densitometry data were obtained as described in Methods.
(TIF)

S7 Fig Effect of sodium butyrate on the distribution of β-catenin. Trophoblasts were incubated with NaCl (control) or NaBu and then stained with anti-beta-catenin antibody and DAPI as described in Methods and Fig 5. Results from two separate experiments are shown. The third experiment is shown in Fig 5.
(TIF)

S8 Fig Expression of Wnt and Wnt receptors. Total RNA was obtained from trophoblast cells using the RNeasy Plus Mini kit (Qiagen, Valencia CA). cDNA synthesized from 1μg of RNA using Superscript II Reverse transcriptase (Invitrogen, Carlsbad CA) was used in PCR reactions using primers shown below. PCR reactions were performed using AccuPower PCR premix (Bioneer, Alameda CA) at an annealing temperature of 60°C.
(TIF)

S9 Fig Effect of LiCl on trophoblast morphology. Cells were incubated in the presence of NaCl (control) or LiCl for 7 days as described in Methods and Fig 6. Each row represents phase contrast images from different cell preparations.
(TIF)

S10 Fig Interaction of trophoblasts with endothelial cells. Cocultures were established as described in Methods and Fig 10. Cells were stained with antibodies against VE-Cadherin (red) and CK7 (green) and nuclei were stained using DAPI (blue). Endothelial cells were incubated with trophoblast conditioned medium (TCM) or Trophoblast medium (TM) as described in Methods and Fig 10. The cells were stained with antibodies against VE-Cadherin (red) and CK7 (green) and nuclei were stained using DAPI (blue). Each row represents different cell preparations. The third preparation is shown in Fig 10.
(TIF)

S11 Fig Effect of TNFalpha and CHIR99201 on trophoblasts. Cells were incubated in the presence of TNFalpha, CHIR, CHIR plus TNF, or DMSO (vehicle control) as described in Methods and Fig 12. Phase contrast images from three different experiments are shown.
(TIF)
