# Supplementary figures and images for: Differential Effects of Sodium Butyrate and Lithium Chloride on Rhesus Monkey Trophoblast Differentiation
Source: PLoS One. 2015 Aug 12;10(8):e0135089. doi: 10.1371/journal.pone.0135089 (PMC4533975; doi:10.1371/journal.pone.0135089)

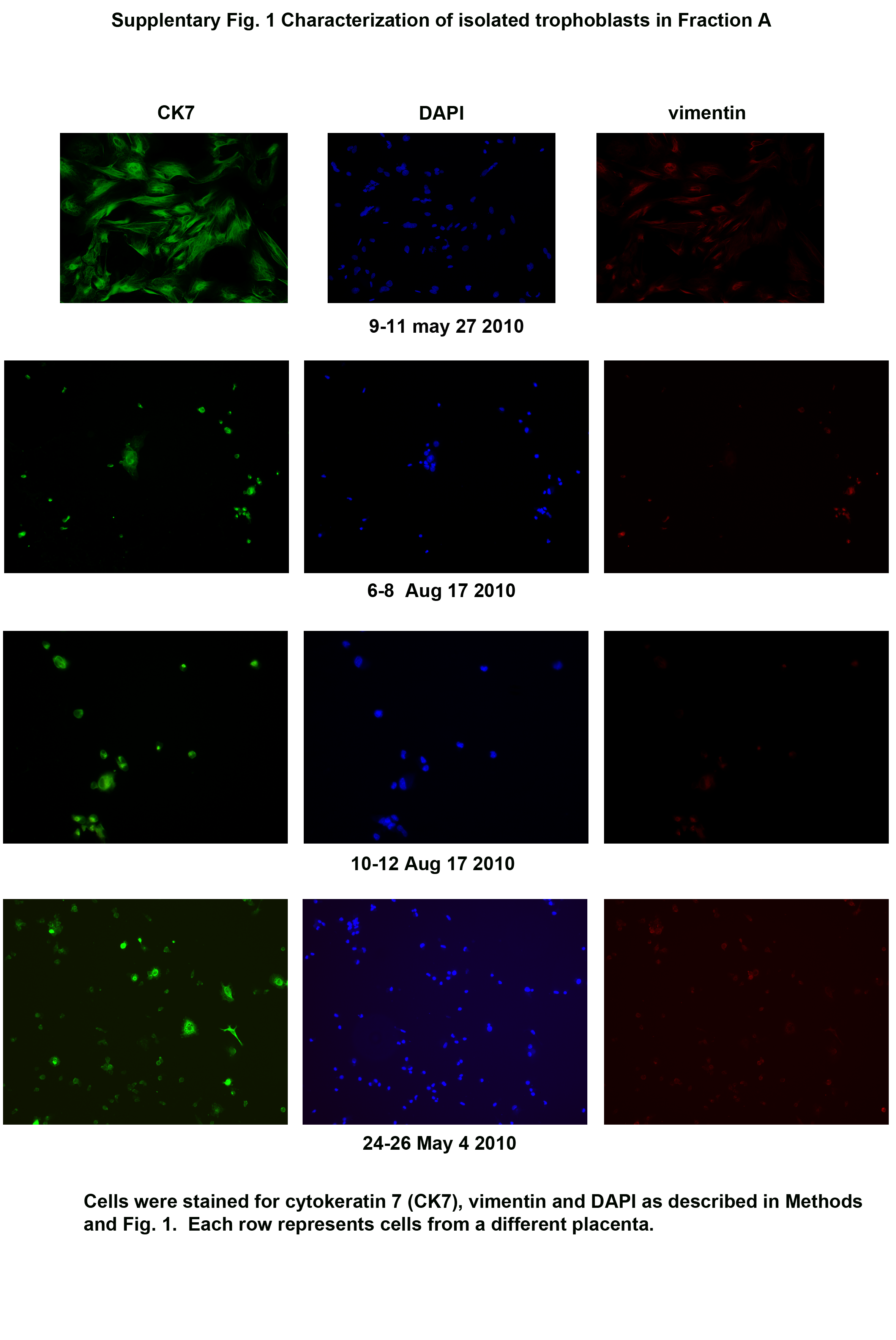

Supplement: S1 Fig — Cells were stained for cytokeratin 7 (CK7), vimentin and DAPI as described in Methods and Fig 1. Each row represents cells from a different placenta. (TIF) [file pone.0135089.s009.tif]

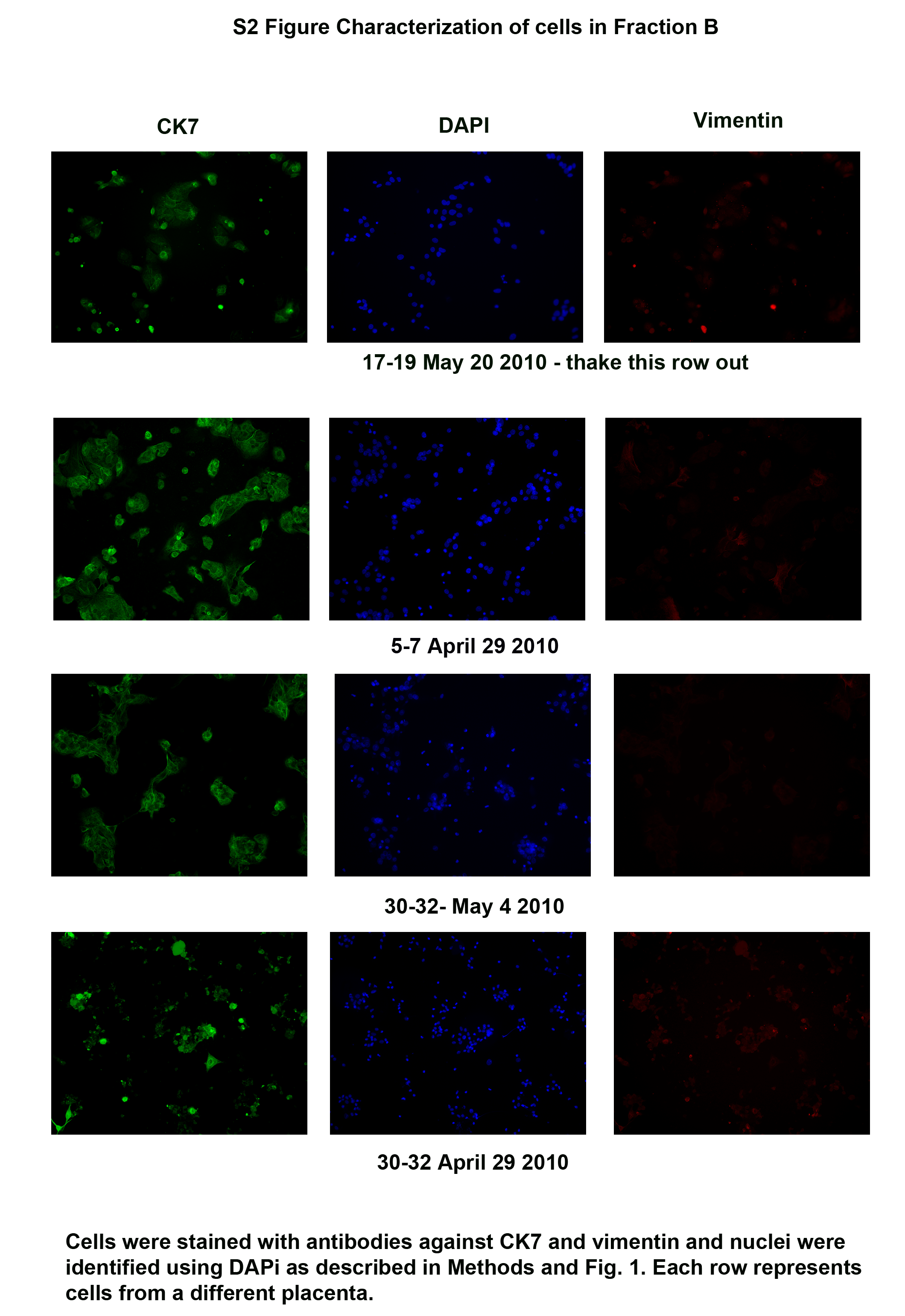

Supplement: S2 Fig — Cells were stained for cytokeratin 7 (CK7), vimentin and DAPI as described in Methods and Fig 1. Each row represents cells from a different placenta. (TIF) [file pone.0135089.s010.tif]

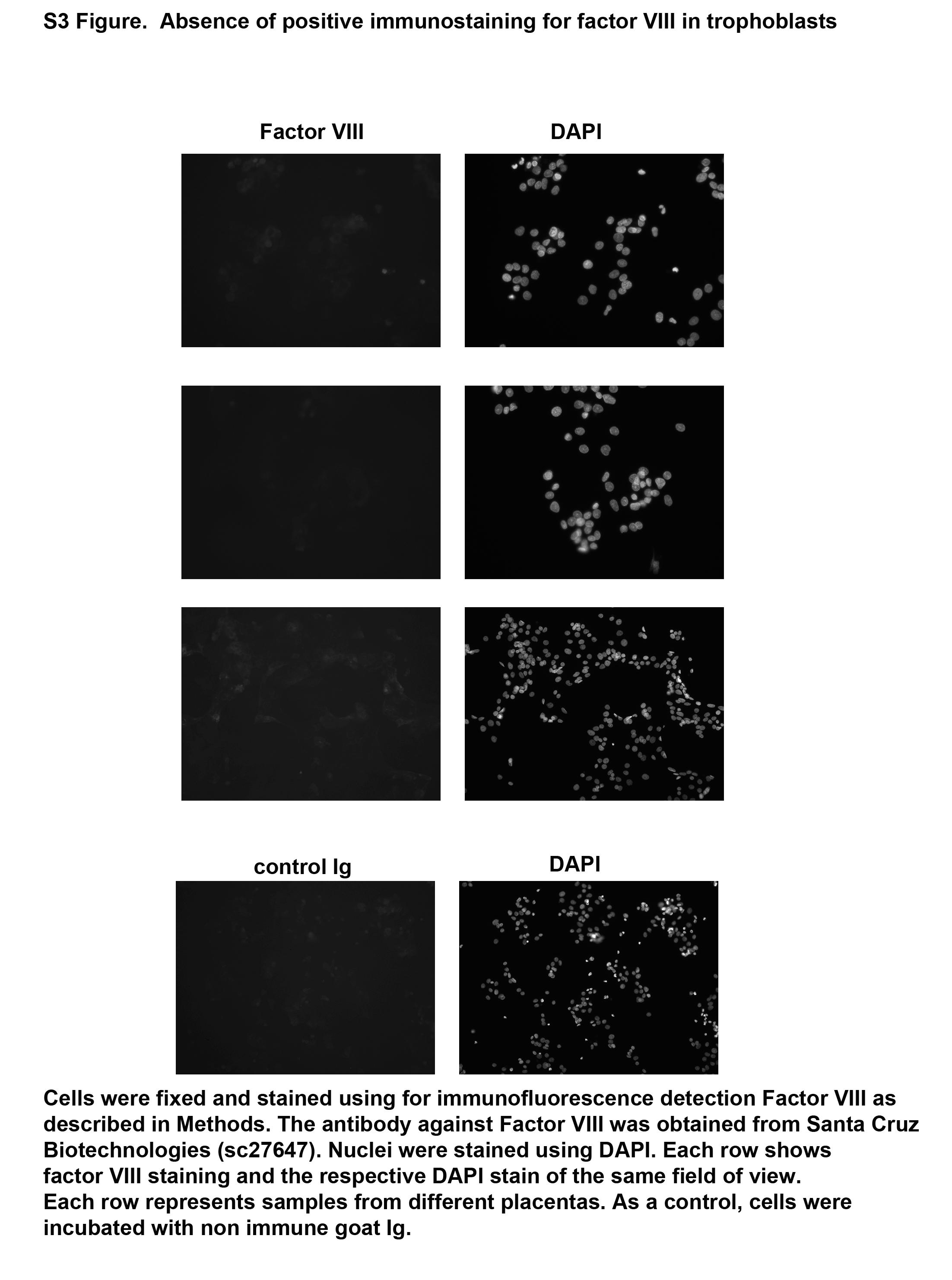

Supplement: S3 Fig — Cells were fixed and stained for immunofluorescence detection of Factor VIII as described in Methods. The antibody against Factor VIII was obtained from Santa Cruz Biotechnologies (sc27647). Nuclei were stained using DAPI. Each row shows factor VIII staining and the respective DAPI stain of the same field of view. Each row represents samples from different placentas. As a control, cells were incubated with non-immune goat Ig. (TIF) [file pone.0135089.s011.tif]

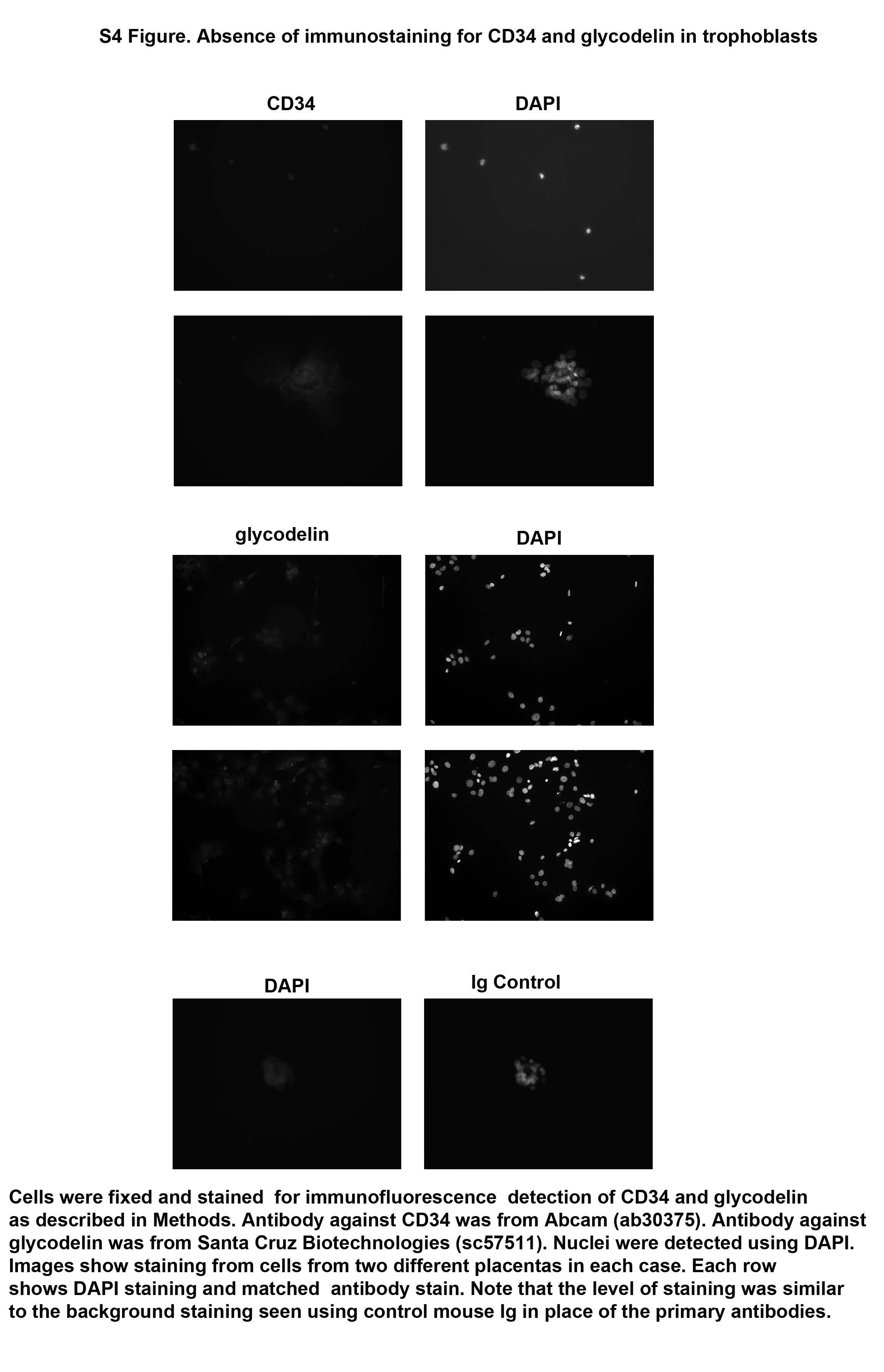

Supplement: S4 Fig — Cells were fixed and stained for immunofluorescence detection of CD34 and glycodelin as described in Methods. Antibody against CD34 was from Abcam (ab30375). Antibody against glycodelin was from Santa Cruz Biotechnologies (sc57511). Nuclei were detected using DAPI. Images show staining from cells from two different placentas in each case. Each row shows DAPI staining and the respective antibody stain. Note that the level of staining was similar to the background staining seen using control mouse Ig in place of the primary antibodies. (TIF) [file pone.0135089.s012.tif]

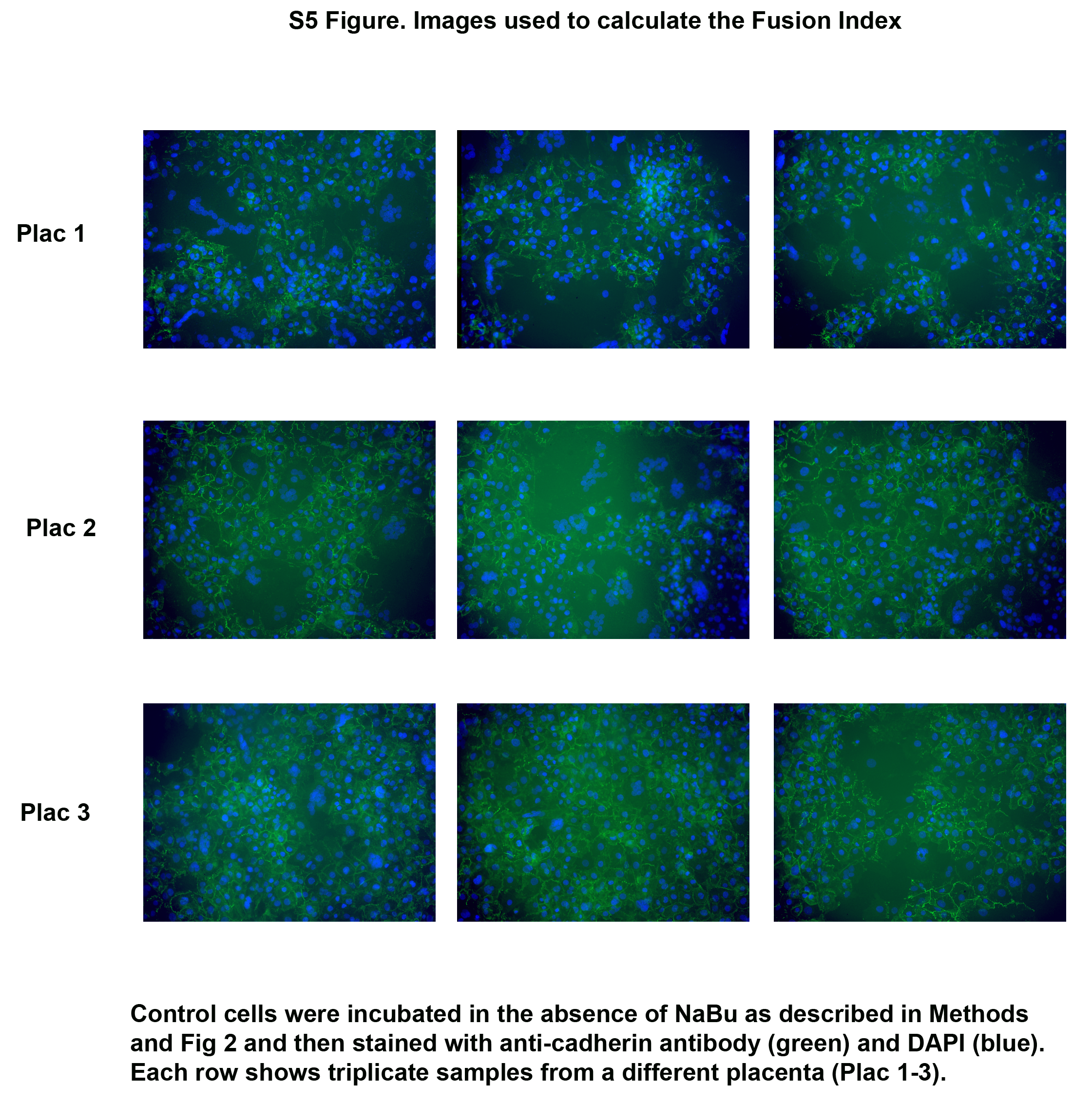

Supplement: S5 Fig — Control cells were incubated in the absence of NaBu as described in Methods and Fig 2 and then stained with anti-cadherin antibody (green) and DAPI (blue). Each row shows triplicate samples from a different placenta (Plac 1–3). (TIF) [file pone.0135089.s013.tif]

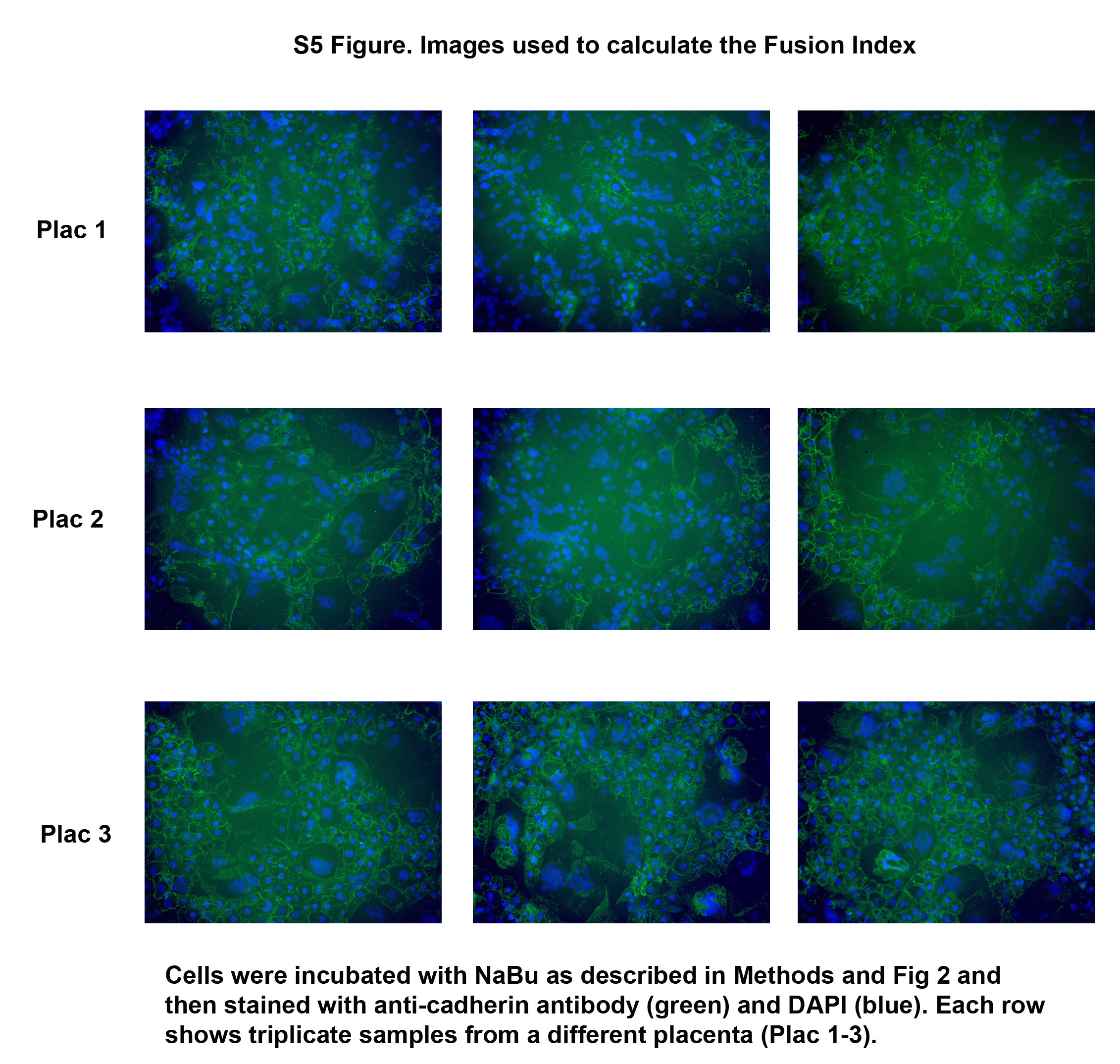

Supplement: S6 Fig — Control cells were incubated with NaBu as described in Methods and Fig 2 and then stained with anti-cadherin antibody (green) and DAPI (blue). Each row shows triplicate samples from a different placenta (Plac 1–3). levels. QPCR data and densitometry data were obtained as described in Methods. (TIF) [file pone.0135089.s014.tif]

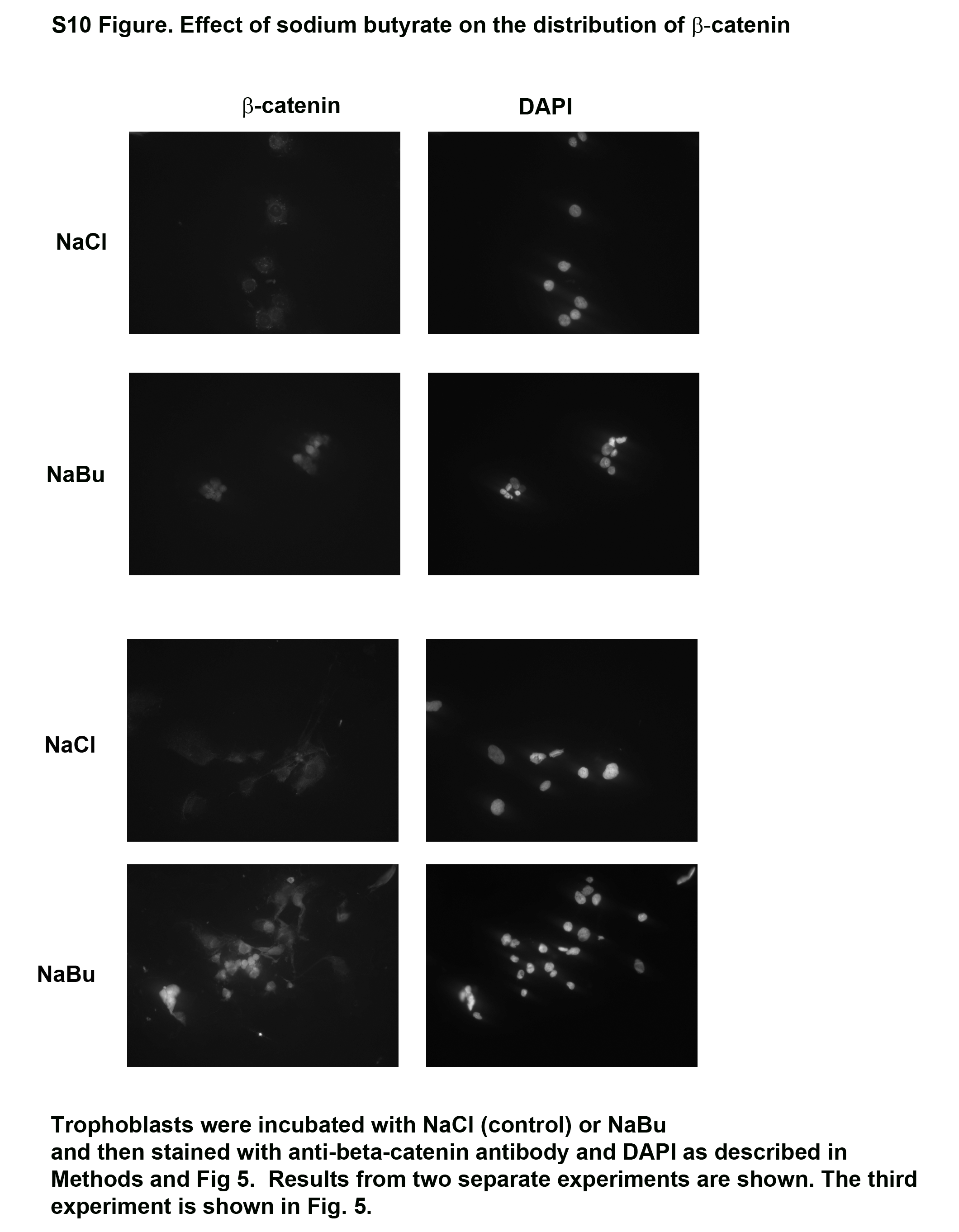

Supplement: S7 Fig — Trophoblasts were incubated with NaCl (control) or NaBu and then stained with anti-beta-catenin antibody and DAPI as described in Methods and Fig 5. Results from two separate experiments are shown. The third experiment is shown in Fig 5. (TIF) [file pone.0135089.s015.tif]

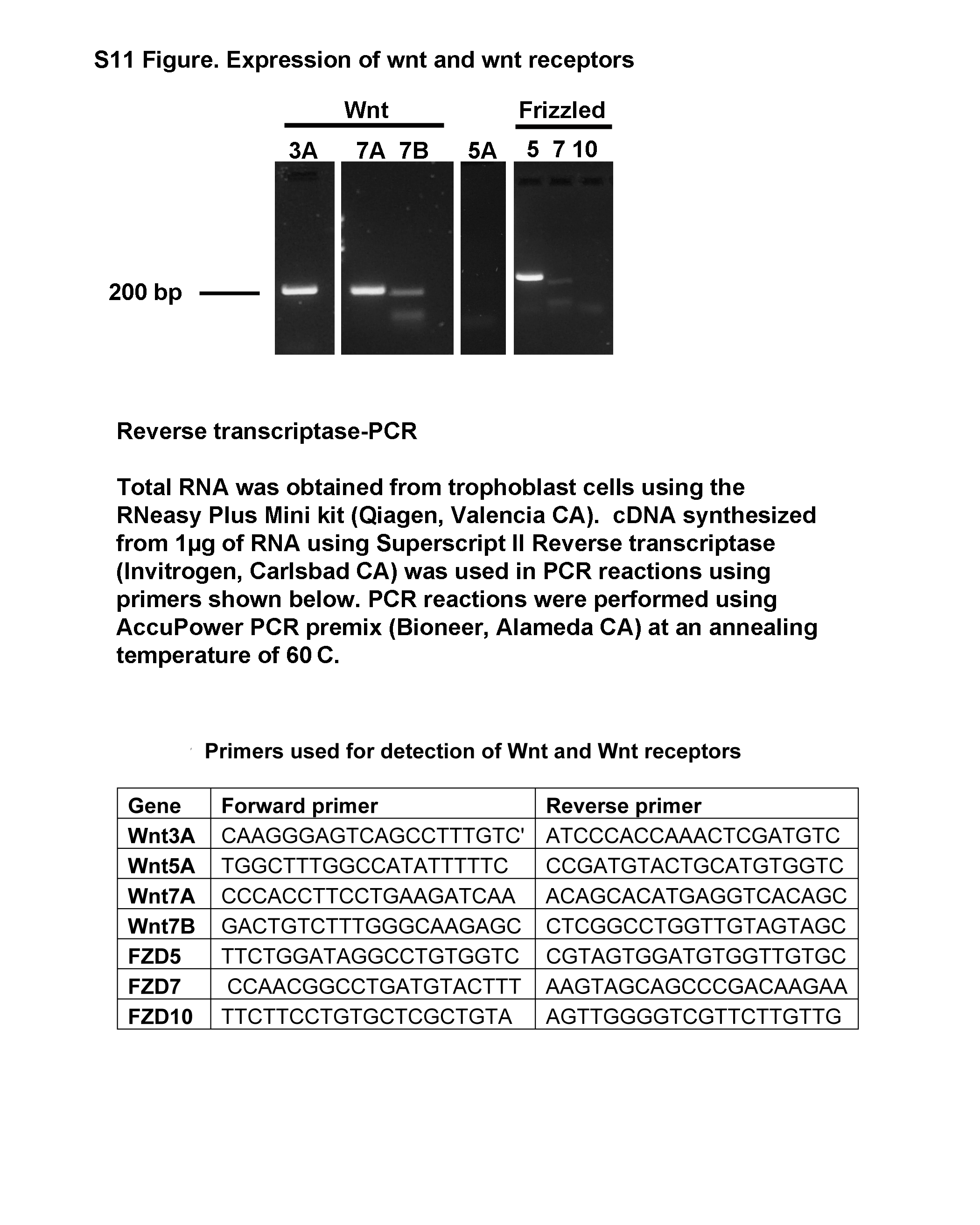

Supplement: S8 Fig — Total RNA was obtained from trophoblast cells using the RNeasy Plus Mini kit (Qiagen, Valencia CA). cDNA synthesized from 1μg of RNA using Superscript II Reverse transcriptase (Invitrogen, Carlsbad CA) was used in PCR reactions using primers shown below. PCR reactions were performed using AccuPower PCR premix (Bioneer, Alameda CA) at an annealing temperature of 60°C. (TIF) [file pone.0135089.s016.tif]

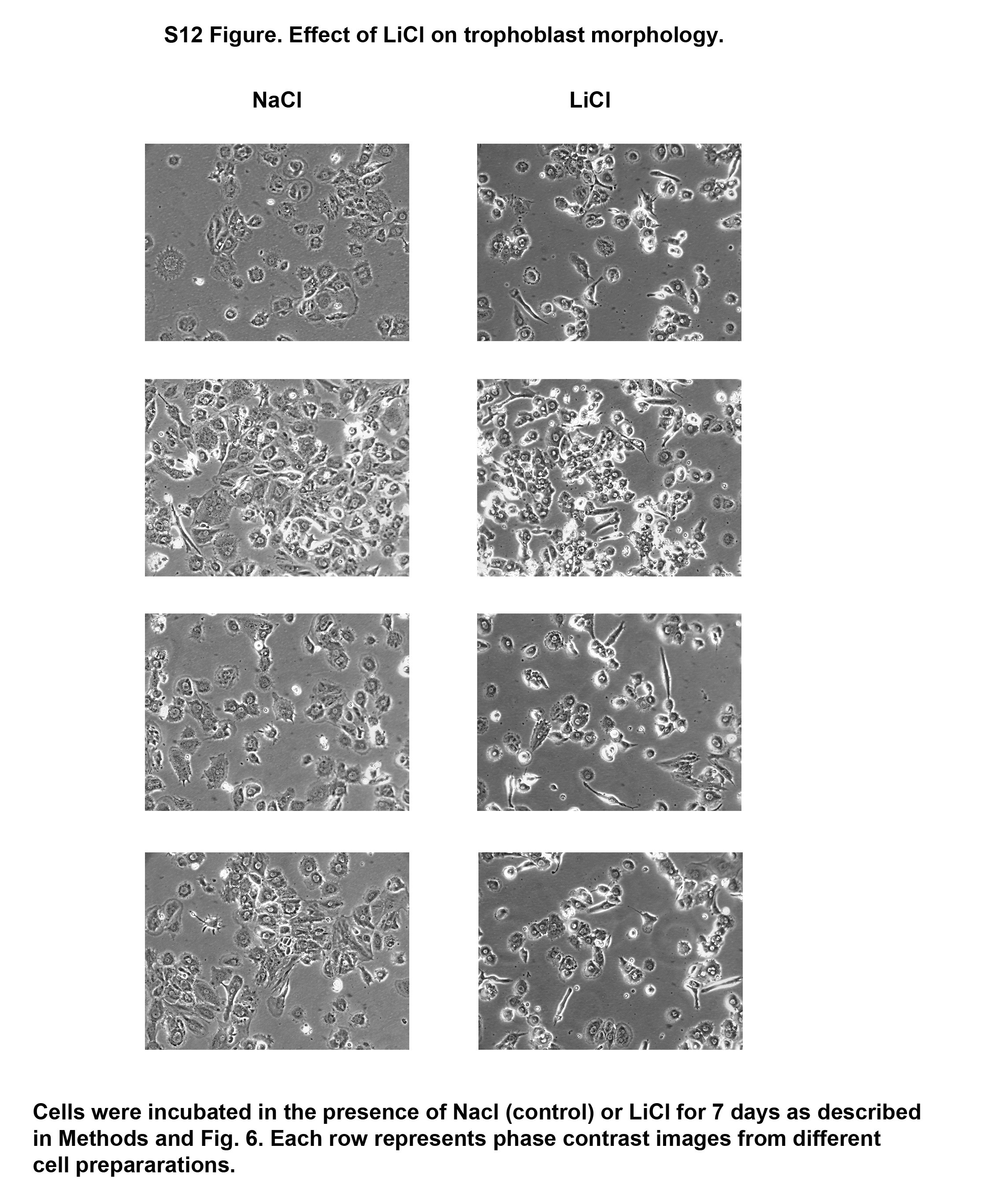

Supplement: S9 Fig — Cells were incubated in the presence of NaCl (control) or LiCl for 7 days as described in Methods and Fig 6. Each row represents phase contrast images from different cell preparations. (TIF) [file pone.0135089.s017.tif]

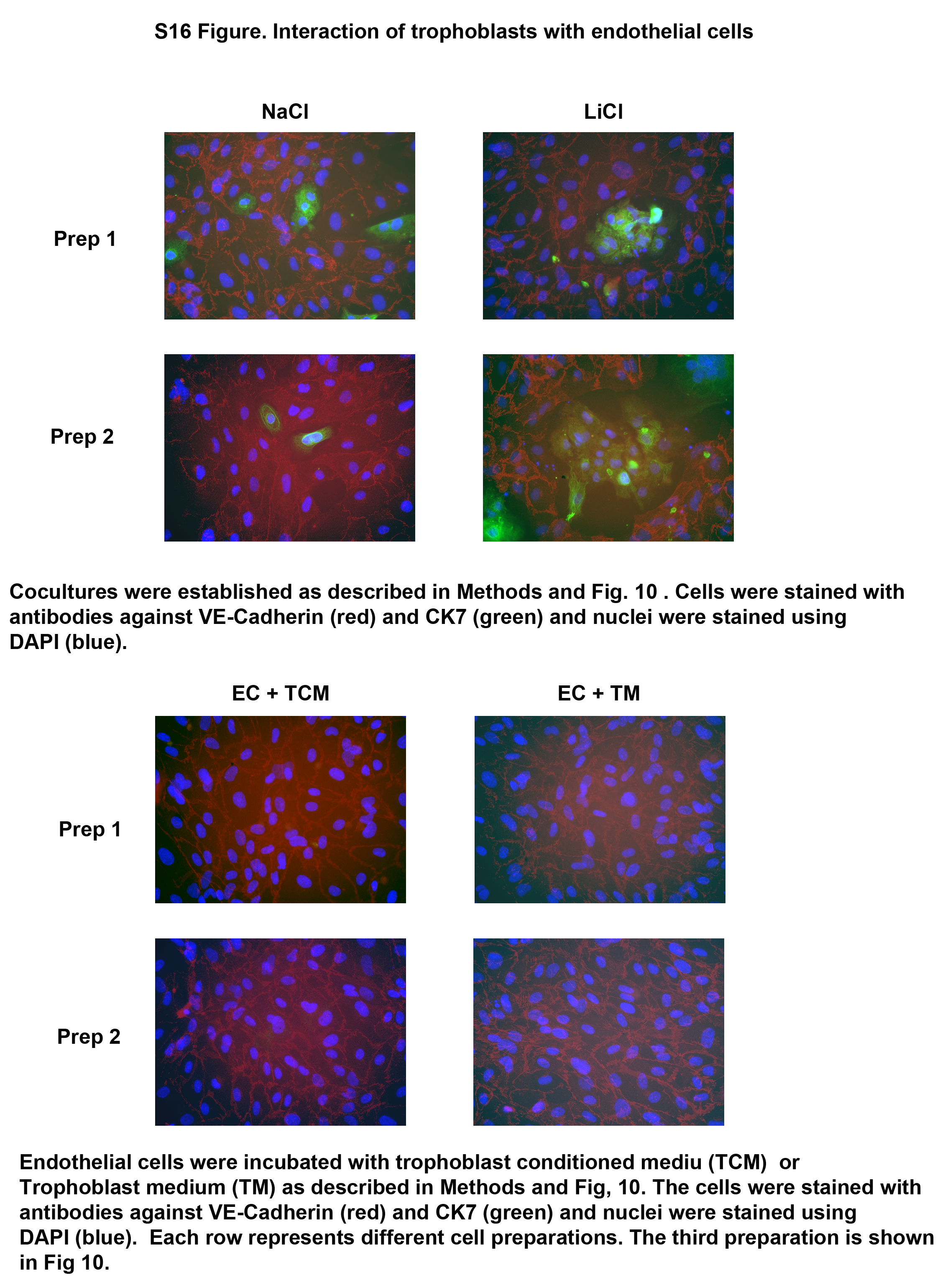

Supplement: S10 Fig — Cocultures were established as described in Methods and Fig 10. Cells were stained with antibodies against VE-Cadherin (red) and CK7 (green) and nuclei were stained using DAPI (blue). Endothelial cells were incubated with trophoblast conditioned medium (TCM) or Trophoblast medium (TM) as described in Methods and Fig 10. The cells were stained with antibodies against VE-Cadherin (red) and CK7 (green) and nuclei were stained using DAPI (blue). Each row represents different cell preparations. The third preparation is shown in Fig 10. (TIF) [file pone.0135089.s018.tif]

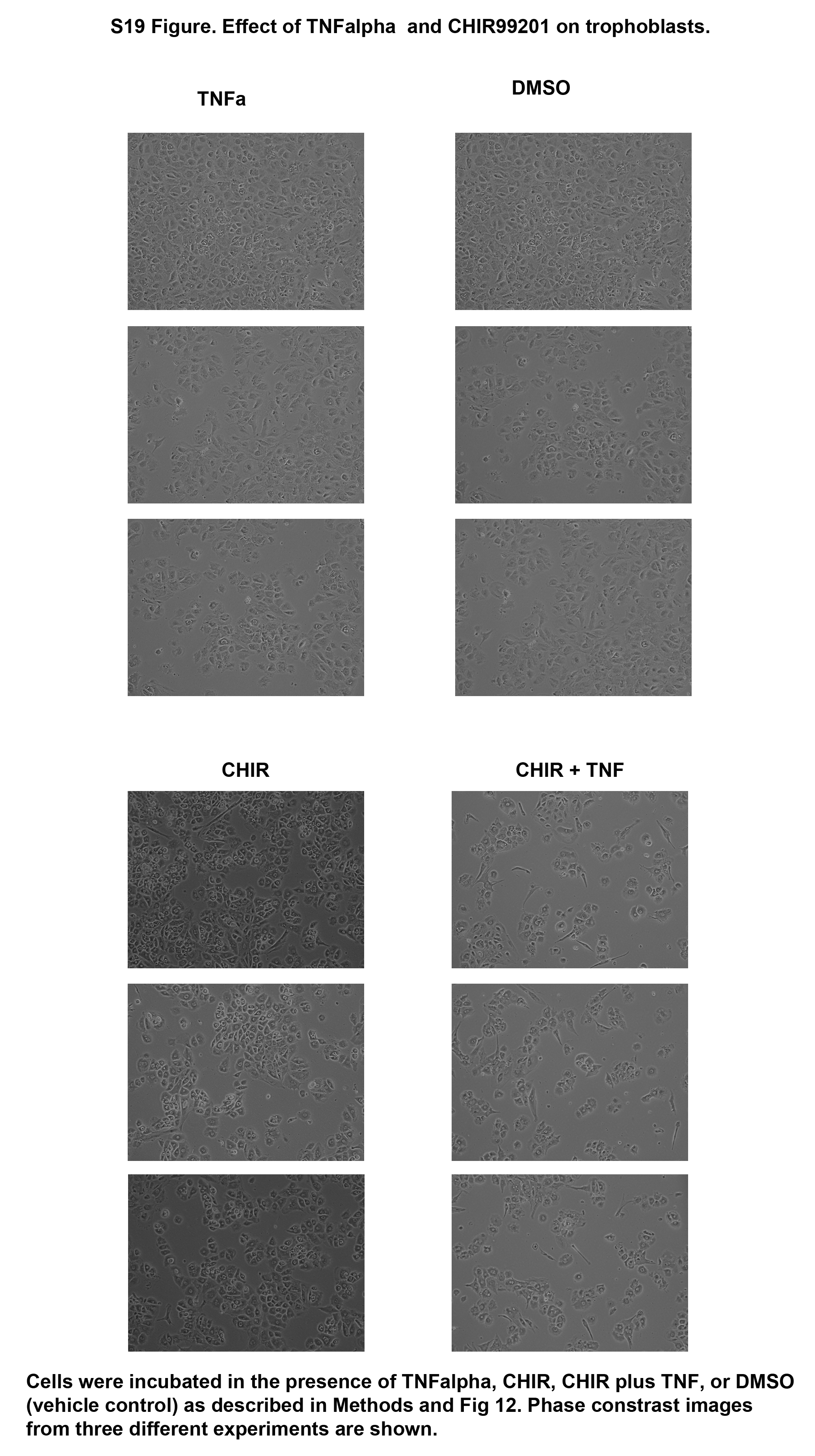

Supplement: S11 Fig — Cells were incubated in the presence of TNFalpha, CHIR, CHIR plus TNF, or DMSO (vehicle control) as described in Methods and Fig 12. Phase contrast images from three different experiments are shown. (TIF) [file pone.0135089.s019.tif]
